# Serum Levels of Adiponectin Are Strongly Associated with Lipoprotein Subclasses in Healthy Volunteers but Not in Patients with Metabolic Syndrome

**DOI:** 10.3390/ijms25095050

**Published:** 2024-05-06

**Authors:** Iva Klobučar, Hansjörg Habisch, Lucija Klobučar, Matias Trbušić, Gudrun Pregartner, Andrea Berghold, Gerhard M. Kostner, Hubert Scharnagl, Tobias Madl, Saša Frank, Vesna Degoricija

**Affiliations:** 1 Department of Cardiology, Sisters of Charity University Hospital Centre, 10000 Zagreb, Croatia; iva.klobucar@gmail.com (I.K.); matias.trbusic@gmail.com (M.T.); 2 Otto Loewi Research Center, Medicinal Chemistry, Medical University of Graz, 8010 Graz, Austria; hansjoerg.habisch@medunigraz.at (H.H.); tobias.madl@medunigraz.at (T.M.); 3 Department of Medicine, University Hospital Centre Osijek, 31000 Osijek, Croatia; klobucar.lucija@gmail.com; 4 School of Medicine, University of Zagreb, 10000 Zagreb, Croatia; vesna.degoricija@mef.hr; 5 Institute for Medical Informatics, Statistics, and Documentation, Medical University of Graz, 8036 Graz, Austria; gudrun.pregartner@medunigraz.at (G.P.); andrea.berghold@medunigraz.at (A.B.); 6 Gottfried Schatz Research Center, Molecular Biology and Biochemistry, Medical University of Graz, 8010 Graz, Austria; gerhard.kostner@medunigraz.at; 7 Clinical Institute of Medical and Chemical Laboratory Diagnostics, Medical University of Graz, 8036 Graz, Austria; hubert.scharnagl@medunigraz.at; 8 BioTechMed-Graz, 8010 Graz, Austria; 9 Department of Medicine, Sisters of Charity University Hospital Centre, 10000 Zagreb, Croatia

**Keywords:** adiponectin, lipoprotein subclasses, nuclear magnetic resonance spectroscopy, metabolic syndrome

## Abstract

Metabolic syndrome (MS) is a widespread disease in developed countries, accompanied, among others, by decreased adiponectin serum levels and perturbed lipoprotein metabolism. The associations between the serum levels of adiponectin and lipoproteins have been extensively studied in the past under healthy conditions, yet it remains unexplored whether the observed associations also exist in patients with MS. Therefore, in the present study, we analyzed the serum levels of lipoprotein subclasses using nuclear magnetic resonance spectroscopy and examined their associations with the serum levels of adiponectin in patients with MS in comparison with healthy volunteers (HVs). In the HVs, the serum levels of adiponectin were significantly negatively correlated with the serum levels of large buoyant-, very-low-density lipoprotein, and intermediate-density lipoprotein, as well as small dense low-density lipoprotein (LDL) and significantly positively correlated with large buoyant high-density lipoprotein (HDL). In patients with MS, however, adiponectin was only significantly correlated with the serum levels of phospholipids in total HDL and large buoyant LDL. As revealed through logistic regression and orthogonal partial least-squares discriminant analyses, high adiponectin serum levels were associated with low levels of small dense LDL and high levels of large buoyant HDL in the HVs as well as high levels of large buoyant LDL and total HDL in patients with MS. We conclude that the presence of MS weakens or abolishes the strong associations between adiponectin and the lipoprotein parameters observed in HVs and disturbs the complex interplay between adiponectin and lipoprotein metabolism.

## 1. Introduction

Overnutrition and a sedentary lifestyle, and thereby associated abdominal obesity, are major promotors of the pathophysiological events that lead to the development of metabolic syndrome (MS) [1,2]. MS is a cluster of pathophysiological conditions that comprises abdominal obesity, insulin resistance, hyperglycemia, and arterial hypertension, as well as dyslipidemia with increased levels of serum triglycerides and decreased levels of high-density lipoprotein (HDL) [3,4,5].

MS dyslipidemia is a consequence of a complex interplay between the inflamed insulin-resistant visceral adipose tissue and insulin-resistant liver, where serum lipases and lipid-transfer proteins play an important role. More specifically, local adipose tissue inflammation triggered by infiltrating macrophages, and the concomitant insulin resistance-driven adipose tissue lipolysis, lead to an oversupply of the liver with free fatty acids. This, in turn, enhances hepatic triglyceride (TG) synthesis and very-low-density lipoprotein (VLDL) release into the circulation. VLDL overproduction and the decreased VLDL catabolism due to the low serum lipoprotein lipase (LPL) activity (secondary to low-grade persistent inflammation and insulin resistance) are the principal causes of the increased serum levels of TG-rich VLDL particles and hypertriglyceridemia in MS (reviewed in [6,7]). In addition to TG-rich VLDL, the increased levels of atherogenic small dense low-density lipoprotein (LDL) as well as low levels of high-density lipoprotein (HDL) are the hallmarks of dyslipidemia in MS. The cholesterol ester transfer protein (CETP)-mediated enrichment of LDL with TGs from the TG-rich VLDL and the subsequent extensive lipolysis of the TG-enriched LDL by hepatic lipase (HL) give rise to the elevated levels of small dense LDL in MS. An accelerated catabolism of the small dense HDL generated by the CETP-mediated TG-enrichment of HDL and the subsequent HL-mediated HDL lipolysis are the major causes for decreased levels of HDL in MS (reviewed in [6,7]).

Adiponectin, a protein secreted by the adipose tissue, circulates as a complex of a low-molecular-weight trimer, a medium-molecular-weight hexamer, and a high-molecular-weight multimer. Adiponectin serum levels are decreased in obesity, insulin resistance, type 2 diabetes, and MS (reviewed in [8,9,10,11,12,13]). Upon binding to its receptors (AdipoR1 and AdipoR2), adiponectin activates multiple signaling pathways which increase glucose utilization and fatty acid oxidation in the skeletal muscle and, by reducing gluconeogenesis and glycogenolysis, decreases hepatic glucose production [4,14,15,16,17,18,19].

Numerous studies have demonstrated a positive relationship between serum adiponectin and the serum levels of HDL and large buoyant LDL, as well as a negative relationship between adiponectin and the large TG-rich VLDL and small dense LDL [20,21,22,23,24,25]. Experiments in cultured cells and mice, as well as kinetic studies in humans, revealed that the positive relationship between adiponectin and HDL reflects an augmenting effect of adiponectin on HDL biogenesis and a diminishing effect on HDL catabolism [9,26,27,28]. Specifically, through the upregulation of the hepatic production of apolipoprotein A-I (apoA-I) and ATP-binding cassette transporter A1 (ABCA1), adiponectin promotes cholesterol efflux, the lipidation of apoA-I, and, in turn, the biogenesis of HDL [29]. On the other hand, adiponectin lowers the serum levels of the TG-rich VLDL via the upregulation of LPL and the downregulation of HL, thus impeding the TG-enrichment of HDL and in turn the HL-mediated generation of the rapidly catabolizing small dense HDL [26,27,28,29,30,31]. Accelerated catabolism of TG-rich VLDL due to the adiponectin-mediated induction of LPL and VLDL receptor expression, along with the adiponectin-induced downregulation of HL, diminishes the conversion of large buoyant to small dense LDL. This interplay between adiponectin, LPL, VLDL receptors, and HL is the principal driver of the negative associations between adiponectin and the large TG-rich VLDL as well as small dense LDL [28,31,32,33].

The relationship between the serum levels of adiponectin and lipoproteins has been extensively studied in healthy children and adults, in obese and lean adolescents, as well as in adults with or without type 2 diabetes (T2D) [8,12,22,23,25,30,34,35,36,37,38,39,40,41,42]. However, no study has examined whether the relationships between the serum levels of adiponectin and the lipoprotein subclasses observed in a healthy state also exist in MS, a pathophysiological condition accompanied by decreased adiponectin levels and perturbed lipoprotein metabolism [11,40]. We hypothesized that the relationship between the serum levels of adiponectin and the lipoprotein subclasses differs between healthy volunteers (HVs) and patients with MS. Therefore, in the present study, we determined the serum levels of adiponectin and lipoprotein subclasses in healthy volunteers (HVs) and patients with MS and examined whether the associations between adiponectin and the serum lipoproteins differ in these groups.

## 2. Results

### 2.1. Demographic and Clinical Characteristics and Laboratory Data in HVs and Patients with MS

The demographic and clinical characteristics of the study groups, as well as the chronic medication of patients with MS, have been described previously [43,44] and are shown in Table 1 and Table S1.

Routine laboratory parameters have also been described previously [43,44] and are shown in Table 2. Adiponectin serum levels were significantly lower in patients with MS compared to HVs (Table 2). Differences in the serum levels of lipoprotein classes and subclasses between the HVs and patients with MS are shown in Table S2.

### 2.2. Correlation Analyses between Adiponectin and the Serum Levels of VLDL, IDL, and LDL

After Bonferroni correction for multiple testing, the serum levels of adiponectin were significantly negatively correlated with the serum levels of cholesterol (VLDL-C), free cholesterol (VLDL-FC), triglycerides (VLDL-TG), phospholipids (VLDL-PL), and apolipoprotein (apo) B (VLDL-apoB) in total VLDL, as well as in the large buoyant VLDL subclasses 1–3 (with the exception of VLDL2-C) in HVs, but not in patients with MS (Table 3).

The serum levels of adiponectin were also significantly negatively correlated with the serum levels of free cholesterol (IDL-FC) and triglycerides (IDL-TG) in intermediate-density lipoprotein (IDL) in HVs but, again, not in patients with MS (Table 4).

We observed significant negative correlations between adiponectin and the serum levels of cholesterol, phospholipids, and apoB in small dense LDL subclasses 4 and 5, as well as free cholesterol and triglycerides in LDL subclass 5 in HVs, but not in patients with MS (Table 5). Interestingly, the serum levels of adiponectin were significantly positively correlated with the serum levels of phospholipids in the large buoyant LDL subclass 1 in patients with MS, but not in the HVs (Table 5).

The significant correlations observed for VLDL, IDL, and LDL in both groups (Table 3, Table 4 and Table 5) were rendered insignificant after adjusting for age, sex, body mass index (BMI), waist circumference, or metabolic syndrome severity score (MetSSS) as well as for age, sex, BMI, and C-reactive protein (CRP) (Tables S3 and S4).

### 2.3. Correlation Analyses between Adiponectin and the Serum Levels of HDL

In the HVs, adiponectin was significantly positively correlated with the serum levels of cholesterol, free cholesterol, phospholipids, and apoA-I in total HDL as well as in large buoyant HDL subclasses 1 and 2 (Table 6). In the HVs, the adiponectin serum levels were additionally significantly positively correlated with the serum levels of triglycerides and apoA-II in large buoyant HDL subclass 1, as well as with the serum levels of phospholipids in HDL subclass 3. In patients with MS, the serum levels of adiponectin were significantly (positively) correlated only with the serum levels of phospholipids in total HDL (Table 6).

After adjusting for age, sex, and BMI (Model 1), the correlations between adiponectin and the serum levels of cholesterol and phospholipids in HDL subclasses 1 and 2 in the HVs remained significant. These significant correlations, with the exception of the correlation between adiponectin and the cholesterol levels in HDL subclass 1, also remained significant after additional adjustment for CRP (Model 2). While only the correlation between adiponectin and the cholesterol levels in HDL subclass 2 remained significant after adjusting for age, sex, and waist circumference (Model 3), adjusting for age, sex, and MetSSS rendered all correlations insignificant (Model 4) (Table 7). The observed significant positive correlation between adiponectin and the serum levels of phospholipids in total HDL in patients with MS was found to be insignificant in all tested adjustment models (Table S4).

### 2.4. Associations of Lipoprotein Parameters with Low and High Adiponectin Levels in HVs and Patients with MS

#### 2.4.1. Orthogonal Partial Least-Squares Discriminant Analysis (OPLS-DA)

**Figure 1:**
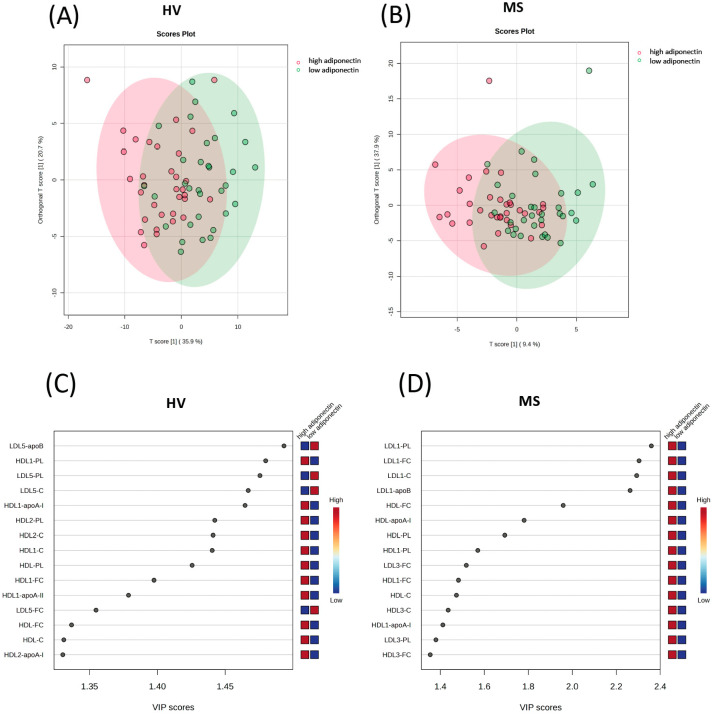
Associations of lipoproteins and their subclasses with low and high adiponectin in HVs and patients with MS. Differences in lipoprotein parameters between HVs with adiponectin < 15.1 µg/mL (low adiponectin) vs. adiponectin ≥ 15.1 µg/mL (high adiponectin), as well as between (**B**) MS patients with adiponectin < 13.0 µg/mL (low adiponectin) vs. adiponectin ≥ 13.0 µg/mL (high adiponectin) were examined through OPLS-DA. The upper panel shows score plots of (**A**) the HVs and (**B**) patients with MS; the lower panel shows the VIP scores of the 15 highest-ranking parameters in (**C**) the HVs and (**D**) patients with MS. apoA-I, apolipoprotein A-I; apoA-II, apolipoprotein A-II; apoB, apolipoprotein B; C, cholesterol; FC, free cholesterol; HDL, high-density lipoprotein; HV, healthy volunteer; LDL, low-density lipoprotein; MS, metabolic syndrome patient; OPLS-DA, orthogonal partial least-squares discriminant analysis; PL, phospholipid; VIP, variable of importance projection.

We performed OPLS-DA to examine the relative associations of the lipoprotein classes and subclasses with low and high adiponectin levels in HVs and patients with MS. We considered adiponectin values below the respective median (HV: 15.1 µg/mL; MS: 13.0 µg/mL) as “low”, and those ≥ 15.1 µg/mL and ≥13.0 µg/mL, respectively, as “high”. Differences in the lipoprotein parameters between HVs and patients with MS with low versus high adiponectin levels are shown in Table S5. Permutation R^2^Y was 0.374 (*p* < 0.001) and 0.374 (*p* = 0.015) and the cross-validation score Q^2^ was 0.274 (*p* < 0.001) and Q^2^ = 0.187 (*p* = 0.001) in HVs and patients with MS, respectively (Figure S1). The OPLS-DA revealed partial clustering of the lipoprotein parameters into high and low adiponectin samples in both HVs and patients with MS (Figure 1A,B). The relative strength of the associations between the lipoprotein parameters and low or high adiponectin levels were determined by the variable of importance projection (VIP) scores, separately in the HVs and patients with MS (the VIP scores of all parameters in the HVs and patients with MS are presented in Table S6).

In both the HVs and patients with MS, the top fifteen lipoprotein parameters with the highest VIP scores comprised various LDL and HDL parameters but none of the VLDL and IDL parameters (Figure 1C,D). The top five parameters in the HVs comprised the indicators of the serum levels of small dense LDL subclass 5 (LDL5-apoB, LDL5-PL, and LDL5-C) as well as those of large buoyant HDL subclass 1 (HDL1-PL and HDL1-apoA-I). In contrast with the HVs, in patients with MS, the top five parameters comprised the indicators of the serum levels of large buoyant LDL subclass 1 (LDL1-PL, LDL1-FC, LDL1-C, and LDL1-apoB), as well as an indicator of total HDL (HDL-FC). In the HVs, the residual parameters within the top fifteen parameters comprised nine parameters that indicate the serum levels of large buoyant HDL particles, as well as one small dense LDL parameter (LDL5-FC). In patients with MS, the residual parameters comprised three indicators of the serum levels of total HDL (HDL-apoA-I, HDL-PL, and HDL-C) and large buoyant HDL subclass 1 (HDL1-PL, HDL1-FC, and HDL1-apoA-I), as well as two indicators of the serum levels of medium-dense HDL (HDL3-C and HDL3-FC) and LDL (LDL3-FC and LDL3-PL) (Figure 1C,D).

#### 2.4.2. Logistic Regression Analysis

**Figure 2:**
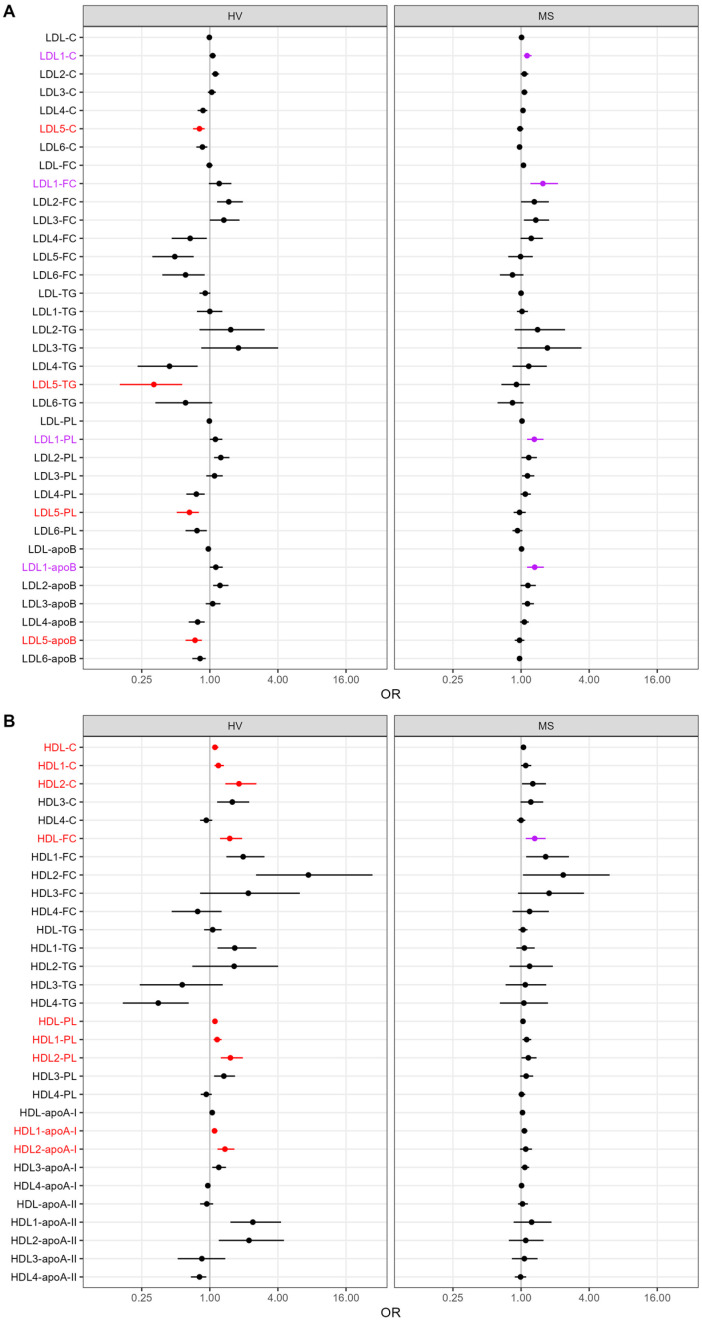
Logistic regression analyses to assess associations of lipoproteins and their subclasses with high vs. low adiponectin levels in the HVs and patients with MS. The results are presented as odds ratios (ORs) and 95% confidence intervals (CI) per increase in one unit (mg/dL) of (**A**) the LDL and (**B**) HDL parameters. The *x*-axis is presented on a log-2 scale. *p*-values < 0.0005 are considered statistically significant after Bonferroni correction for multiple testing. Significant parameters as well as their respective ORs and 95% CIs are depicted in red. In the MS patients, the parameters, ORs, and 95% CIs depicted in purple are those with the highest VIP scores in the OPLS-DA. apoA-I, apolipoprotein A-I; apoA-II, apolipoprotein A-II; apoB, apolipoprotein B; C, cholesterol; CI, FC, free cholesterol; HDL, high-density lipoprotein; HV, healthy volunteer; LDL, low-density lipoprotein; MS, metabolic syndrome patient; OPLS-DA, orthogonal partial least-squares discriminant analysis; PL, phospholipid; VIP, variable of importance projection.

To further examine the associations of the lipoprotein subclasses with high adiponectin levels, we performed univariable logistic regression analyses. In line with the results obtained with OPLS-DA, none of the VLDL and IDL parameters were significantly associated with high adiponectin levels (Figure S2). Again, in accordance with the OPLS-DA, in the HVs, there were significant associations of adiponectin with several indicators of small dense LDL subclass 5 (negatively), as well as with total and large buoyant HDL subclasses 1 and 2 (positively) (Figure 2A,B and Table S7). In patients with MS, there were no significant associations after Bonferroni correction for multiple testing. However, the lowest p-values were observed for the four indicators of the large buoyant LDL subclass 1 (Figure 2A and Table S7) as well as an indicator of total HDL (Figure 2B and Table S7). These parameters also showed the five highest VIP scores in the OPLS-DA (Figure 1D). All significant associations observed in the univariable logistic regression analysis were found to be insignificant upon adjusting for age, sex, and BMI (not shown).

## 3. Discussion

Herein, we show for the first time that the associations between adiponectin and the lipoprotein subclasses observed in HVs are much weaker in patients with MS and that lipoprotein subclasses associated with low vs. high adiponectin levels are different in HVs and patients with MS. Low-grade persistent inflammation and insulin resistance, perturbed lipoprotein metabolism with decreased HDL and increased levels of the atherogenic TG-enriched VLDL of very large size, as well as diminished adiponectin bioavailability [6,8,11,39,40,45], are likely causes of the weakening and disruption of the associations between adiponectin and lipoproteins in MS. Interestingly, adiponectin was significantly negatively correlated with the indicators of adipose tissue mass, namely BMI and waist circumference, in the HVs but not in the patients with MS, and was furthermore not correlated with the indicators of inflammation (C-reactive protein or interleukin-6) in either study group (Table S8). Notably, the correlations between lipoproteins and clinical and laboratory parameters also differed between the HVs and patients with MS (Tables S9–S13). These findings suggest different regulations of adiponectin and lipoproteins in MS than in a healthy state, which might be, at least in part, responsible for the lack or weakening of the associations between adiponectin and the lipoproteins in patients with MS. Kinetic studies in humans have revealed the very complex nature of the relationship between adiponectin and lipoprotein metabolism, which undergoes interrelated regulation. This is exemplified by the strong relationships between the fractional catabolic rate of HDL-apoA-I and adiponectin as well as VLDL-apoB, whereby the production rate of VLDL-apoB has been found to be determined by total plasma apoA-II, in a process partially mediated by adiponectin [27,30,42].

In the present study, the negative associations between adiponectin and the large buoyant VLDL in HVs are in accordance with the adiponectin-mediated induction of LPL and VLDL receptors, important determinants of VLDL catabolism [28,31,32,33]. By decreasing the release of free fatty acids from adipocytes and by increasing the uptake of free fatty acids by the skeletal muscle, as well as by decreasing the hepatic expression of HL, adiponectin diminishes the supply of the liver with free fatty acids and, as a consequence, the production of VLDL [31,46,47]. The negative associations between adiponectin and the large buoyant VLDL as well as small dense LDL observed in HVs, but not in the patients with MS, are in accordance with the results of previous studies examining the relationship between adiponectin and the lipoprotein subclasses in healthy adults [23,25], as well as in obese and lean adolescents [35] and patients with T2D [38].

The VLDL- and HL-lowering activities of adiponectin [28,31,32,33] and, as a consequence, the impeded remodeling of the large buoyant to small dense LDL are likely causes of the observed negative associations between adiponectin and the small dense LDL subclasses 4 and 5 in HVs. The absence of these associations in patients with MS might be due to the impaired regulation of LPL and HL by adiponectin, as well as the decreased serum levels of adiponectin and the increased serum levels of large TG-enriched VLDL, which give rise to small dense LDL [6,39,40,45].

In the present study, the serum levels of adiponectin were positively associated with the serum levels of apoA-I and apoA-II carried by large buoyant HDL, which most likely reflects the established positive effects of adiponectin on HDL bioavailability through the promotion of biogenesis and attenuation of the catabolism of HDL [27,30,48]. Considering that HDL particles may contain either apoA-I, apoA-II, or both apoA-I and apoA-II [49,50], the observed positive correlations between adiponectin and the serum levels of apoA-I and apoA-II in the large buoyant HDL subclass 1 most likely reflect a positive relationship between adiponectin and the large buoyant HDL particles that contain both apolipoproteins. In contrast, the observed positive correlation between adiponectin and the serum levels of apoA-I, but not apoA-II, in HDL subclass 2 most likely reflects the involvement of the subclass that contains only apoA-I.

The associations between adiponectin and HDL were much weaker in patients with MS. It is conceivable that the negative impact of MS pathophysiology on LPL, which plays an important role in the biogenesis of HDL [33], or other molecular players involved in HDL metabolism [8,9], weakens the relationship between adiponectin and HDL in MS. The positive associations between adiponectin and the large buoyant HDL particles in the HVs observed in the present study are in accordance with previous reports [22,35]. However, previous studies also found significant negative associations between adiponectin and small dense HDL particles, which, in the present study, were only weakly and, after Bonferroni correction for multiple testing, not statistically significantly associated with adiponectin. This discrepancy might be due to the different methodologies used for the determination of the HDL subclasses, namely isolation and analyses of HDL subclasses via ultracentrifugation and gradient gel electrophoresis in the previous study [22] vs. NMR spectroscopy in the present study or due to the differences in the characteristics of the study participants, namely, lean and obese adolescents in the previous study [35] vs. healthy adults in the present study.

In the present study, the significant associations between adiponectin and some HDL parameters were the only associations that remained significant after adjusting for age, sex, and BMI or waist circumference. This is in line with the previously reported independent associations between adiponectin and HDL, but not VLDL, IDL, and LDL [38]. These findings imply that confounders which modulate the bioavailability of adiponectin and lipoproteins [8,11,38,51,52,53], markedly modulate the associations of adiponectin with VLDL, IDL, and LDL, but less profoundly modulate the associations of adiponectin with HDL. A profound impact of adiponectin on the biogenesis and catabolism of HDL [27,30,48] might be responsible for the persistent associations between adiponectin and HDL in the present and previous study [38].

It is well established that obesity, insulin resistance, and T2D modulate the serum levels of both lipoprotein subclasses and adiponectin [8,12]. It is also well known that lipoprotein subclasses markedly differ in their pro- and anti-atherogenic activities, with profound pro-atherogenic capacity of small dense LDL and large buoyant VLDL and the anti-atherogenic activity of HDL noted [54,55,56,57]. In the present study, we observed a positive association of adiponectin with the anti-atherogenic lipoprotein profile, exemplified by a negative association between adiponectin and atherogenic small dense LDL, as well as a positive association with anti-atherogenic HDL, and large buoyant LDL, which exhibits low atherogenicity [54]. Considering the association of adiponectin with a favorable lipoprotein profile, and the fact that adiponectin, in contrast with the lipoprotein subclasses, can be easily measured in each clinical laboratory, it is tempting to assume that the classification of subjects/patients according to their adiponectin levels into low/high adiponectin level groups might complement, or even replace, lipoproteins as estimators of atherogenic risk. Additionally, boosting adiponectin expression through lifestyle-driven or pharmacological treatments may help achieve a favorable lipoprotein profile and thus reduce atherogenic risk.

The major strength of our study is the detailed analysis of the serum levels of lipoprotein subclasses in HVs and patients with MS, which enabled a comprehensive analysis of the associations between the lipoprotein parameters and adiponectin in the study groups. However, due to the design of the present study, we were only able to examine associations but not causality for the relationships between adiponectin and the lipoprotein subclasses. Accordingly, the mechanistic relationship between adiponectin and the lipoprotein subclasses could not be examined. Considering the rather moderate number of available samples in the study groups, our results will need to be confirmed in larger cohorts of HVs and patients with MS. Furthermore, the fact that the study population was exclusively of European descent limits the generalizability of the study results.

Based on our results, we conclude that the presence of MS weakens the strong associations between adiponectin and the lipoprotein parameters observed in HVs. Accordingly, it appears that the complex molecular regulatory network, comprising adiponectin and other molecular players involved in the regulation of lipoprotein metabolism, operates differently under physiological conditions than in the presence of insulin resistance, inflammation, and perturbed metabolism, the pathophysiological constellations encountered in MS.

## 4. Materials and Methods

### 4.1. Study Design, Participants, and Routine Laboratory Procedures

We performed a cross-sectional investigation of demographic, clinical, and laboratory blood serum parameters in 65 healthy volunteers and 65 individuals with metabolic syndrome, aged 45 to 65 years, who did not suffer from any other concomitant acute or chronic diseases. Metabolic syndrome was defined according to the joint statement given by multiple international professional societies in 2009 [3], with the chosen population-specific normal waist circumference thresholds of <102 cm for men and <88 cm for women. The inclusion and exclusion criteria, as well as all study procedures, are described in detail in our previous reports [43,44]. The study was approved by the local ethics committees of the Sisters of Charity University Hospital Centre, Zagreb, Croatia (EP 13125/17-4); the University of Zagreb, School of Medicine, Croatia; and the Medical University of Graz, Austria (31-532 ex 18/19)**.** All participants signed an informed consent form, and the study was performed in accordance with the principles of Good Clinical Practice Guidelines and the Declaration of Helsinki [58].

### 4.2. Adiponectin Measurements

Adiponectin was measured using a latex-enhanced turbidimetric immunoassay (Lot#230222) (Denka Co. Ltd., Tokyo, Japan) on a Beckman Coulter AU680 analyzer (Beckman Coulter, Krefeld, Germany). The coefficients of variation were 2.0% and 0.9% at levels of 2.0 and 7.7 µg/mL, respectively. The latex assay was compared with a human adiponectin ELISA kit (Lot#3940441) (Millipore via Sigma-Aldrich, Vienna, Austria) by running in parallel 51 sera with a wide range of adiponectin concentrations. Both assays resulted in comparable values and correlated very well. The acceptance criteria were slope 1.0 ± 0.1 and R ≥ 0.9.

### 4.3. Lipoprotein Profiling Using Nuclear Magnetic Resonance (NMR) Spectroscopy

The serum lipoprotein subclasses were measured on a Bruker 600 MHz Avance Neo NMR spectrometer (Bruker, Rheinstetten, Germany) using the Bruker IVDr lipoprotein subclass analysis protocol, as described previously [43,44].

### 4.4. Statistics

Qualitative variables were described using absolute and relative frequencies. Depending on the data distribution, the quantitative variables were summarized using the mean and standard deviations (SD) or medians and interquartile ranges (q1, q3). Differences in the variable values between the HVs and patients with MS were assessed using Fisher’s exact test, a *t*-test, or the Mann–Whitney U test. Correlation analyses using Spearman’s correlation coefficient were performed separately for the HVs and patients with MS. The impact of confounders on the associations between adiponectin and lipoproteins was examined via partial correlation analyses using four models: Model 1—age, sex, and BMI; Model 2—age, sex, BMI, and CRP; Model 3—age, sex, waist circumference; Model 4—age, sex, and MetSSS. MetSSS was calculated according to the method of Wiley and Carrington [59].

Furthermore, OPLS-DA [60] was used to determine the relative associations between the lipoprotein classes/subclasses and high or low adiponectin levels, separately in the HVs and patients with MS. These analyses, the associated data consistency checks, and 7-fold cross-validation [61] were performed using MetaboAnalyst (R package “ropls”) [62,63].

Associations between lipoproteins and high/low adiponectin levels were also examined through univariable and multivariable logistic regression analysis. In the multivariable analyses, we adjusted for age, sex, and BMI. The results are presented as odds ratios (OR) and 95% confidence intervals (CI).

A *p*-value of <0.05 was considered significant for the analyses regarding differences in the demographic and clinical characteristics and standard laboratory data, as well as correlation analyses between adiponectin and the clinical and laboratory parameters. However, when assessing differences in the serum levels of the lipoprotein subclasses between the study groups, for the correlation analyses of adiponectin with the lipoprotein subclasses and the logistic regression analyses, Bonferroni correction (0.05/95) was applied to correct for multiple testing, and thus, a *p*-value of < 0.0005 was considered significant. R version 4.1.0 was used for all analyses except for the OPLS-DA.

## Figures and Tables

**Table 1:** Differences in demographic and clinical characteristics between HVs and patients with MS.

Variable	All (*N* = 130)	HV (*N* = 65)	MS (*N* = 65)	*p*
Age (years)	56.0 (50.0, 60.0)	56.0 (50.0, 59.0)	57.0 (50.0, 60.0)	0.440
Sex (female)	62 (47.7%)	31 (47.7%)	31 (47.7%)	1.000
Body weight (kg)	87.5 (75.2, 102.8)	77.0 (68.0, 88.0)	98.0 (86.0, 113.5)	**<0.001**
Body height (m)	1.74 ± 0.10	1.75 ± 0.10	1.73 ± 0.11	0.243
BMI (kg/m^2^)	28.8 (25.1, 32.7)	25.1 (23.7, 28.1)	32.6 (29.8, 35.9)	**<0.001**
Waist circumference (cm)	103.1 ± 16.5	92.2 ± 11.6	113.9 ± 13.2	**<0.001**
SBP (mmHg)	130.0 (120.0, 140.0)	120.0 (115.0, 130.0)	140.0 (130.0, 145.0)	**<0.001**
DBP (mmHg)	80.0 (70.0, 80.0)	70.0 (70.0, 80.0)	80.0 (70.0, 80.0)	**<0.001**
MAP (mmHg)	96.7 (88.3, 100.0)	88.3 (85.0, 96.7)	98.3 (96.7, 101.7)	**<0.001**
MetSSS	2.4 (0.8, 4.2)	0.7 (0.0, 2.3)	3.8 (2.6, 4.8)	**<0.001**
**Chronic diseases**				
Arterial hypertension	60 (46.2%)	0 (0.0%)	60 (92.3%)	**<0.001**
Diabetes mellitus type 2	27 (20.8%)	0 (0.0%)	27 (41.5%)	**<0.001**
Stable angina pectoris	2 (1.5%)	0 (0.0%)	2 (3.1%)	0.496
Atrial fibrillation	2 (1.5%)	0 (0.0%)	2 (3.1%)	0.496
CVI and TIA	1 (0.8%)	0 (0.0%)	1 (1.5%)	1.000
Intermittent claudications	4 (3.1%)	0 (0.0%)	4 (6.2%)	0.119
Deep venous thrombosis	6 (4.6%)	1 (1.5%)	5 (7.7%)	0.208
Pulmonary embolism	2 (1.5%)	0 (0.0%)	2 (3.1%)	0.496
**Functions and habits**				
Smoking	34 (26.2%)	16 (24.6%)	18 (27.7%)	0.842
Physical activity (≥3 times/week)	105 (80.8%)	58 (89.2%)	47 (72.3%)	**0.025**
Menstrual cycle (female)	18/62 (29.0%)	12/31 (38.7%)	6/31 (19.4%)	0.161

Data are presented as *n* (%), mean ± standard deviation, or median (q1, q3). Differences between HVs and patients with MS were tested using Fisher’s exact test, a *t*-test, or the Mann–Whitney U test. *p*-values < 0.05 are considered statistically significant and are depicted in bold. BMI, body mass index; cm, centimeter; CVI, cerebrovascular infarction; DBP, diastolic blood pressure; HV, healthy volunteer; kg, kilogram; m, meter; MAP, mean arterial pressure; MS, metabolic syndrome patient; MetSSS, metabolic syndrome severity score; *N*, number; SBP, systolic blood pressure; TIA, transitory ischemic attack.

**Table 2:** Differences in laboratory data between HVs and patients with MS.

Variable	All (*N* = 130)	HV (*N* = 65)	MS (*N* = 65)	*p*
Adiponectin (µg/mL)	14.0 (10.4, 19.4)	15.1 (11.6, 22.2)	13.0 (9.9, 17.2)	**0.006**
Triglycerides (mmol/L)	1.3 (0.9, 1.9)	1.0 (0.8, 1.4)	1.6 (1.1, 2.2)	**<0.001**
Total cholesterol (mmol/L)	5.3 (4.7, 6.1)	5.5 (5.1, 6.0)	5.0 (4.3, 6.2)	0.057
LDL-C (mmol/L)	3.2 (2.5, 3.7)	3.3 (2.8, 3.7)	3.0 (2.3, 3.7)	0.077
HDL-C (mmol/L)	1.4 (1.1, 1.7)	1.6 (1.4, 1.8)	1.2 (1.0, 1.4)	**<0.001**
Glucose (mmol/L)	5.3 (4.9, 5.7)	4.9 (4.8, 5.2)	5.7 (5.3, 6.5)	**<0.001**
Protein (g/L)	73.0 (70.0, 76.0)	72.0 (69.0, 75.0)	75.0 (71.0, 77.0)	**0.002**
Albumin (g/L)	48.0 (46.0, 49.0)	47.0 (46.0, 49.0)	48.0 (45.0, 49.0)	0.465
CRP (µg/mL)	1.8 (0.8, 3.7)	1.2 (0.6, 2.3)	2.4 (1.2, 5.5)	**<0.001**
IL-6 (pg/mL)	3.0 (2.1, 5.3)	2.3 (1.7, 3.0)	4.1 (2.7, 6.8)	**<0.001**
Bilirubin (µmol/L)	8.5 (6.0, 11.6)	9.6 (7.4, 13.3)	7.4 (5.5, 10.4)	**0.012**
AST (U/L)	23.0 (20.0, 27.0)	23.0 (20.0, 25.0)	23.0 (19.0, 32.0)	0.244
ALT (U/L)	24.0 (19.0, 36.0)	22.0 (18.0, 29.0)	30.0 (22.0, 43.0)	**<0.001**
AP (U/L)	61.0 (51.0, 73.0)	60.0 (49.0, 70.0)	65.0 (52.0, 81.0)	0.065
GGT (U/L)	24.5 (15.2, 38.0)	16.0 (13.0, 30.0)	31.0 (21.0, 44.0)	**<0.001**
CK (U/L)	124.5 (83.0, 186.8)	115.0 (81.0, 153.0)	133.0 (86.0, 226.0)	**0.048**
LDH (U/L)	172.0 (150.5, 192.0)	168.0 (147.0, 191.0)	176.0 (158.0, 193.0)	0.365
Urea (mmol/L)	5.3 (4.5, 6.3)	5.0 (4.2, 6.0)	5.6 (4.8, 6.5)	**0.004**
Urate (µmol/L)	297.5 (249.9, 345.1)	273.7 (232.0, 327.2)	315.3 (279.7, 362.9)	**<0.001**
Creatinine (µmol/L)	77.9 (67.3, 87.6)	77.9 (69.0, 89.4)	76.6 (65.5, 87.0)	0.414
eGFR (mL/min/1.73 m^2^)	88.0 (78.0, 97.1)	87.5 (77.2, 93.6)	88.9 (79.1, 98.0)	0.358
Sodium (mmol/L)	139.0 (138.0, 141.0)	140.0 (138.0, 141.0)	139.0 (138.0, 140.0)	**0.041**
Potassium (mmol/L)	4.2 (4.1, 4.6)	4.3 (4.1, 4.5)	4.2 (4.1, 4.6)	0.703
Chloride (mmol/L)	100.0 (98.2, 102.8)	101.0 (99.0, 103.0)	100.0 (98.0, 101.0)	**0.006**

Data are presented as the median (q1, q3). Differences between the HVs and patients with MS were tested using the Mann–Whitney U test. *p*-values < 0.05 are considered statistically significant and are depicted in bold. LDL-C and eGFR data were available for 60 and 64 patients with MS, respectively. ALT, alanine aminotransferase; AP, alkaline phosphatase; AST, aspartate aminotransferase; CK, creatine kinase; CRP, C-reactive protein; eGFR, estimated glomerular filtration rate; g, gram; GGT, gamma-glutamyl transpeptidase; HV, healthy volunteer; HDL-C, high-density lipoprotein cholesterol; IL-6, interleukin 6; L, liter; LDH, lactate dehydrogenase; LDL-C, low-density lipoprotein cholesterol; m, meter; µg, microgram; min, minute; mL, milliliter; µmol, micromole; mmol, millimole; MS, metabolic syndrome patient; *N*, number; pg, picogram; U, unit.

**Table 3:** Correlation analyses of the serum levels of adiponectin with the serum levels of lipids and apoB in total VLDL and the VLDL subclasses, performed separately for HVs and patients with MS.

	Adiponectin (µg/mL)			
	HV (*N* = 65)		MS (*N* = 65)	
Variable (mg/dL)	r	*p*	r	*p*
VLDL-C	−0.44	**0.0003**	−0.15	0.2406
VLDL1-C	−0.44	**0.0002**	−0.18	0.1565
VLDL2-C	−0.42	0.0005	−0.16	0.2099
VLDL3-C	−0.45	**0.0002**	−0.15	0.2334
VLDL4-C	−0.37	0.0021	−0.09	0.4980
VLDL5-C	0.02	0.8480	0.19	0.1281
VLDL-FC	−0.44	**0.0003**	−0.15	0.2456
VLDL1-FC	−0.52	**<0.0001**	−0.22	0.0724
VLDL2-FC	−0.44	**0.0002**	−0.21	0.0932
VLDL3-FC	−0.43	**0.0004**	−0.19	0.1381
VLDL4-FC	−0.37	0.0025	−0.08	0.5392
VLDL5-FC	0.02	0.8595	−0.02	0.8983
VLDL-TG	−0.51	**<0.0001**	−0.23	0.0625
VLDL1-TG	−0.53	**<0.0001**	−0.27	0.0305
VLDL2-TG	−0.48	**0.0001**	−0.21	0.0957
VLDL3-TG	−0.44	**0.0003**	−0.18	0.1401
VLDL4-TG	−0.40	0.0010	−0.17	0.1778
VLDL5-TG	0.03	0.8010	0.05	0.6917
VLDL-PL	−0.47	**0.0001**	−0.22	0.0847
VLDL1-PL	−0.55	**<0.0001**	−0.26	0.0335
VLDL2-PL	−0.49	**<0.0001**	−0.22	0.0778
VLDL3-PL	−0.45	**0.0001**	−0.21	0.0942
VLDL4-PL	−0.42	0.0005	−0.14	0.2809
VLDL5-PL	−0.10	0.4176	0.07	0.5612
VLDL-apoB	−0.44	**0.0002**	−0.18	0.1623

Spearman’s correlation analyses were used to evaluate the associations of the serum levels of adiponectin with the serum levels of VLDL. *p*-values < 0.0005 are considered statistically significant after Bonferroni correction for multiple testing and are depicted in bold. apoB, apolipoprotein B; C, cholesterol; FC, free cholesterol; HV, healthy volunteer; MS, metabolic syndrome patient; PL, phospholipid; r, Spearman’s correlation coefficient; TG, triglyceride, VLDL, very-low-density lipoprotein.

**Table 4:** Correlation analyses of the serum levels of adiponectin with the serum levels of lipids and apoB in IDL, performed separately for HVs and patients with MS.

	Adiponectin (µg/mL)			
	HV (*N* = 65)		MS (*N* = 65)	
Variable (mg/dL)	r	*p*	r	*p*
IDL-C	−0.42	0.0005	−0.07	0.5765
IDL-FC	−0.43	**0.0003**	−0.09	0.4883
IDL-TG	−0.43	**0.0004**	−0.16	0.1988
IDL-PL	−0.41	0.0006	−0.11	0.3826
IDL-apoB	−0.39	0.0015	−0.08	0.5405

Spearman’s correlation analyses were used to evaluate the associations of the serum levels of adiponectin with the serum levels of IDL. *p*-values < 0.0005 are considered statistically significant after Bonferroni correction for multiple testing. Serum levels of lipids and apoB in IDL are given in mg/dL. apoB apolipoprotein B; C, cholesterol; FC, free cholesterol; HV, healthy volunteer; IDL, intermediate-density lipoprotein; MS, metabolic syndrome patient; PL, phospholipid; r, Spearman’s correlation coefficient; TG, triglyceride.

**Table 5:** Correlation analyses of the serum levels of adiponectin with the serum levels of LDL subclasses, performed separately for HVs and patients with MS.

	Adiponectin (µg/mL)			
	HV (*N* = 65)		MS (*N* = 65)	
Variable (mg/dL)	r	*p*	r	*p*
LDL-C	−0.17	0.1788	0.14	0.2695
LDL1-C	0.18	0.1421	0.40	0.0011
LDL2-C	0.29	0.0175	0.16	0.2032
LDL3-C	0.05	0.6650	0.24	0.0579
LDL4-C	−0.44	**0.0002**	0.15	0.2264
LDL5-C	−0.58	**<0.0001**	−0.05	0.7202
LDL6-C	−0.39	0.0012	−0.19	0.1245
LDL-FC	−0.07	0.5977	0.19	0.1221
LDL1-FC	0.17	0.1826	0.39	0.0012
LDL2-FC	0.33	0.0072	0.19	0.1389
LDL3-FC	0.18	0.1521	0.28	0.0226
LDL4-FC	−0.33	0.0075	0.22	0.0854
LDL5-FC	−0.52	**<0.0001**	0.04	0.7578
LDL6-FC	−0.33	0.0076	−0.14	0.2724
LDL-TG	−0.19	0.1251	0.03	0.7852
LDL1-TG	0.00	0.9788	0.16	0.2032
LDL2-TG	0.16	0.2017	0.16	0.2084
LDL3-TG	0.17	0.1729	0.22	0.0752
LDL4-TG	−0.37	0.0021	0.09	0.4614
LDL5-TG	−0.47	**0.0001**	−0.10	0.4094
LDL6-TG	−0.17	0.1709	−0.17	0.1716
LDL-PL	−0.14	0.2730	0.21	0.0993
LDL1-PL	0.20	0.1185	0.42	**0.0004**
LDL2-PL	0.32	0.0090	0.20	0.1074
LDL3-PL	0.08	0.5141	0.27	0.0328
LDL4-PL	−0.46	**0.0001**	0.16	0.2024
LDL5-PL	−0.59	**<0.0001**	−0.02	0.9014
LDL6-PL	−0.32	0.0083	−0.17	0.1644
LDL-apoB	−0.26	0.0384	0.09	0.4987
LDL1-apoB	0.21	0.0956	0.40	0.0010
LDL2-apoB	0.29	0.0182	0.16	0.2153
LDL3-apoB	0.04	0.7334	0.25	0.0444
LDL4-apoB	−0.49	**<0.0001**	0.14	0.2495
LDL5-apoB	−0.58	**<0.0001**	−0.07	0.5689
LDL6-apoB	−0.41	0.0007	−0.18	0.1539

Spearman’s correlation analyses were used to evaluate the associations between the serum levels of adiponectin and the serum levels of LDL. *p*-values < 0.0005 are considered statistically significant after Bonferroni correction for multiple testing and are depicted in bold. apoB, apolipoprotein B; C, cholesterol; FC, free cholesterol; HV, healthy volunteer; LDL, low-density lipoprotein; MS, metabolic syndrome patient; PL, phospholipid; r, Spearman’s correlation coefficient; TG, triglyceride.

**Table 6:** Correlation analyses of the serum levels of adiponectin with the serum levels of the HDL subclasses, performed separately for the HVs and patients with MS.

	Adiponectin (µg/mL)			
	HV (*N* = 65)		MS (*N* = 65)	
Variable (mg/dL)	r	*p*	r	*p*
HDL-C	0.53	**<0.0001**	0.32	0.0099
HDL1-C	0.58	**<0.0001**	0.27	0.0304
HDL2-C	0.57	**<0.0001**	0.38	0.0018
HDL3-C	0.40	0.0009	0.34	0.0057
HDL4-C	−0.15	0.2239	0.13	0.3129
HDL-FC	0.55	**<0.0001**	0.42	0.0005
HDL1-FC	0.52	**<0.0001**	0.32	0.0087
HDL2-FC	0.47	**0.0001**	0.33	0.0065
HDL3-FC	0.20	0.1123	0.37	0.0027
HDL4-FC	−0.14	0.2639	0.26	0.0385
HDL-TG	0.16	0.1958	0.24	0.0508
HDL1-TG	0.44	**0.0002**	0.30	0.0136
HDL2-TG	0.18	0.1434	0.23	0.0685
HDL3-TG	−0.09	0.4864	0.10	0.4067
HDL4-TG	−0.42	0.0005	0.00	0.9834
HDL-PL	0.59	**<0.0001**	0.43	**0.0004**
HDL1-PL	0.61	**<0.0001**	0.35	0.0042
HDL2-PL	0.59	**<0.0001**	0.40	0.0011
HDL3-PL	0.42	**0.0004**	0.36	0.0030
HDL4-PL	−0.11	0.3640	0.20	0.1132
HDL-apoA-I	0.49	**<0.0001**	0.37	0.0022
HDL1-apoA-I	0.58	**<0.0001**	0.33	0.0082
HDL2-apoA-I	0.54	**<0.0001**	0.31	0.0110
HDL3-apoA-I	0.38	0.0021	0.32	0.0084
HDL4-apoA-I	−0.22	0.0760	0.19	0.1328
HDL-apoA-II	−0.06	0.6577	0.12	0.3386
HDL1-apoA-II	0.46	**0.0001**	0.20	0.1069
HDL2-apoA-II	0.36	0.0031	0.14	0.2698
HDL3-apoA-II	−0.08	0.5036	0.13	0.2986
HDL4-apoA-II	−0.37	0.0027	0.00	0.9822

Spearman’s correlation analyses were used to evaluate the associations between the serum levels of adiponectin and the serum levels of the HDL subclasses. *p*-values < 0.0005 are considered statistically significant after Bonferroni correction for multiple testing and are depicted in bold. ApoA-I, apolipoprotein A-I; apoA-II, apolipoprotein A-II; C, cholesterol; FC, free cholesterol; HV, healthy volunteer; HDL, high-density lipoprotein; MS, metabolic syndrome patient; PL, phospholipid; r, Spearman’s correlation coefficient; TG, triglyceride.

**Table 7:** Partial correlation analyses of the serum levels of adiponectin with the selected serum levels of the HDL subclasses in the HVs.

	Adiponectin (µg/mL)							
	Model 1		Model 2		Model 3		Model 4	
Variable (mg/dL)	r	*p*	r	*p*	r	*p*	r	*p*
HDL-C	0.42	0.0007	0.42	0.0009	0.40	0.0015	0.34	0.0064
HDL1-C	0.44	**0.0004**	0.43	0.0005	0.42	0.0007	0.41	0.0009
HDL2-C	0.45	**0.0003**	0.45	**0.0003**	0.44	**0.0004**	0.39	0.0015
HDL-FC	0.40	0.0012	0.40	0.0015	0.38	0.0023	0.33	0.0080
HDL1-FC	0.38	0.0025	0.37	0.0030	0.35	0.0049	0.34	0.0076
HDL2-FC	0.33	0.0088	0.34	0.0077	0.31	0.0134	0.27	0.0334
HDL1-TG	0.19	0.1425	0.19	0.1515	0.17	0.1811	0.21	0.1045
HDL-PL	0.43	0.0006	0.43	0.0006	0.40	0.0012	0.36	0.0042
HDL1-PL	0.45	**0.0002**	0.45	**0.0003**	0.43	0.0005	0.43	0.0005
HDL2-PL	0.44	**0.0004**	0.44	**0.0003**	0.43	0.0006	0.40	0.0014
HDL3-PL	0.28	0.0283	0.28	0.0267	0.27	0.0366	0.21	0.1004
HDL-apoA-I	0.32	0.0110	0.32	0.0129	0.29	0.0225	0.24	0.0648
HDL1-apoA-I	0.41	0.0009	0.41	0.0011	0.39	0.0017	0.39	0.0016
HDL2-apoA-I	0.37	0.0027	0.37	0.0031	0.35	0.0057	0.32	0.0124
HDL1-apoA-II	0.28	0.0281	0.28	0.0319	0.25	0.0462	0.29	0.0231

Spearman’s partial correlation analyses were used to evaluate the associations of the serum levels of adiponectin with the serum levels of HDL, while accounting for covariates. *p*-values < 0.0005 are considered statistically significant after Bonferroni correction for multiple testing and are depicted in bold. Model 1: Adjusted for age, sex, and BMI. Model 2: Adjusted for age, sex, BMI, and CRP. Model 3: Adjusted for age, sex, and waist circumference. Model 4: Adjusted for age, sex, and MetSSS. ApoA-I, apolipoprotein A-I, apoA-II, apolipoprotein A-II; BMI, body mass index; C, cholesterol; CRP, C-reactive protein; FC, free cholesterol; HDL, high-density lipoprotein; HV, healthy volunteer; MetSSS, metabolic syndrome severity score; PL, phospholipid; r, Spearman’s correlation coefficient; TG, triglyceride.

## Data Availability

Data are available within the article and Supplemental Materials. Raw data are available upon request.

## Supplementary Materials

The following supporting information can be downloaded at: https://www.mdpi.com/article/10.3390/ijms25095050/s1.
