# Supplementary material for: Serum Levels of Adiponectin Are Strongly Associated with Lipoprotein Subclasses in Healthy Volunteers but Not in Patients with Metabolic Syndrome
Source: Int J Mol Sci. 2024 May 6;25(9):5050. doi: 10.3390/ijms25095050 (PMC11084877; doi:10.3390/ijms25095050)
Supplement: Supplementary file 1 [file ijms-25-05050-s001.zip › Figure S1.pdf]

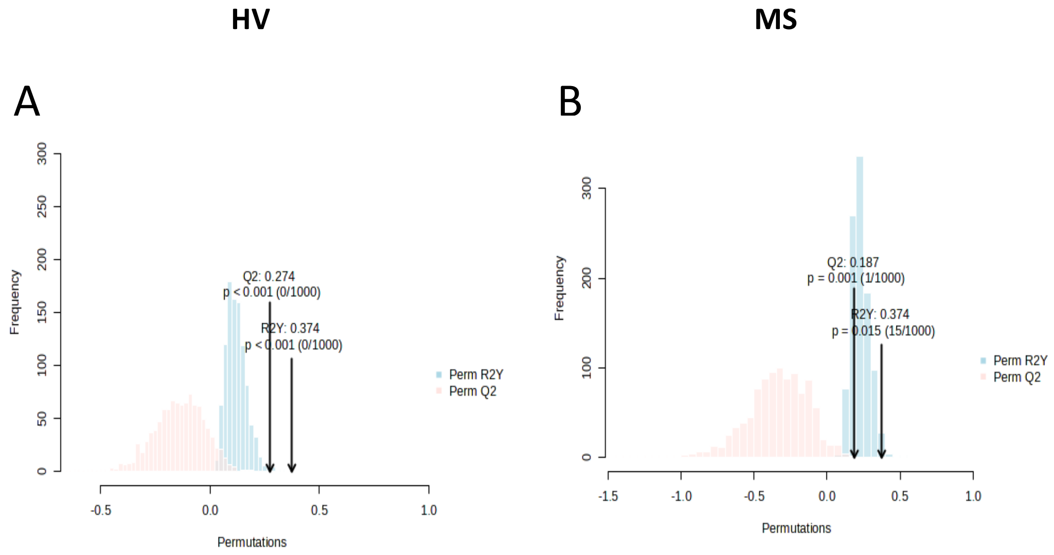

Figure S1. Permutation validity test of OPLS-DA analysis in (A) HVs and (B) patients with MS. OPLS-DA, orthogonal partial least squares discriminant analysis.
