# Supplementary material for: Serum Levels of Adiponectin Are Strongly Associated with Lipoprotein Subclasses in Healthy Volunteers but Not in Patients with Metabolic Syndrome
Source: Int J Mol Sci. 2024 May 6;25(9):5050. doi: 10.3390/ijms25095050 (PMC11084877; doi:10.3390/ijms25095050)
Supplement: Supplementary file 1 [file ijms-25-05050-s001.zip › Figure S2.pdf]

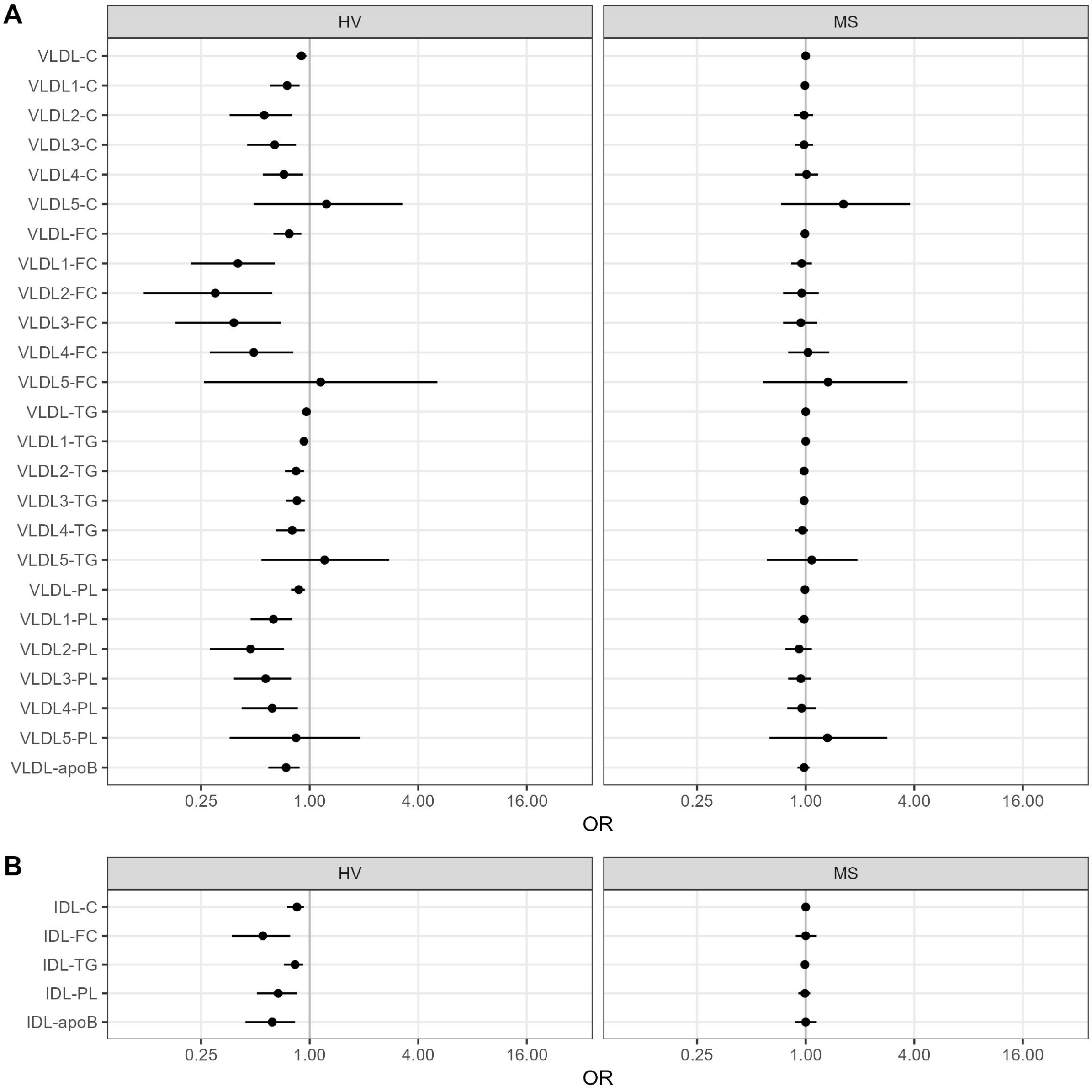

Figure S2. Logistic regression analyses to assess associations of lipoproteins and their subclasses with high vs. low adiponectin in HVs and patients with MS. Results are presented as odds ratios (ORs) and 95% confidence intervals (CI) per increase in one unit (mg/dL) of (A) VLDL and (B) IDL parameters. The x-axis is presented on a log-2 scale. HVs with adiponectin <15.1 µg/mL belong to low (N=33) and those with adiponectin ≥15.1 µg/mL to high adiponectin group (N=32). Patients with MS with adiponectin <13.0 µg/mL belong to low (N=33) and those with adiponectin ≥13.0 µg/mL to high adiponectin group (N=32). *p*-values < 0.0005 are considered statistically significant after a Bonferroni correction for multiple testing. No significant associations were observed. ApoB, apolipoprotein B; C, cholesterol; FC, free cholesterol; HV, healthy volunteer; IDL, intermediate-density lipoprotein; MS, metabolic syndrome patient; OPLS-DA, orthogonal partial least squares discriminant analysis; PL, phospholipid; TG, triglyceride; VLDL, very low-density lipoprotein.
