# Supplementary material for: Serum Levels of Adiponectin Are Strongly Associated with Lipoprotein Subclasses in Healthy Volunteers but Not in Patients with Metabolic Syndrome
Source: Int J Mol Sci. 2024 May 6;25(9):5050. doi: 10.3390/ijms25095050 (PMC11084877; doi:10.3390/ijms25095050)
Supplement: Supplementary file 1 [file ijms-25-05050-s001.zip › Table S1.pdf]

Table S1. Chronic medication in patients with MS.

Drug	MS (N=65)
Metformin	23 (35.4%)
Sulfonylurea	6 (9.2%)
Insulin	5 (7.7%)
Thiazolidinediones	3 (4.6%)
GLP1-agonist	2 (3.1%)
SGLT2-inhibotor	8 (12.3%)
Statin	23 (35.3%)
Fibrates	2 (3.1%)
Omega-3 fatty acids	3 (4.6%)
Perindopril	23 (35.4%)
Ramipril	13 (20.0%)
Trandolapril	1 (1.5%)
Lisinopril	3 (4.6%)
Angiotensin II antagonists	6 (9.2%)
Calcium channel blockers	23 (35.4%)
Nebivolol	6 (9.2%)
Other ß-blockers	17 (13.1%)
Moxonidine	8 (12.3%)
Urapidil	1 (1.5%)
Diuretics	25 (38.5)

Data are presented as n (%). GLP1; glucagon-like peptide 1; MS, metabolic syndrome: SGLT2, sodium-glucose transporter 2.
