# Supplementary material for: Serum Levels of Adiponectin Are Strongly Associated with Lipoprotein Subclasses in Healthy Volunteers but Not in Patients with Metabolic Syndrome
Source: Int J Mol Sci. 2024 May 6;25(9):5050. doi: 10.3390/ijms25095050 (PMC11084877; doi:10.3390/ijms25095050)
Supplement: Supplementary file 1 [file ijms-25-05050-s001.zip › Table S10.pdf]

Table S10. Correlation analyses of serum levels of lipoprotein classes and subclasses with waist circumference, performed separately for the HVs and patients with MS.

Waist circumference (cm)				
	HV (N=65)		MS (N=65)	
**Variable (mg/dL)**	**r**	**p-value**	**r**	**p-value**
**VLDL**				
VLDL-C	0.26	0.0349	-0.13	0.2922
VLDL1-C	0.31	0.0115	-0.16	0.2132
VLDL2-C	0.23	0.0700	-0.15	0.2296
VLDL3-C	0.20	0.1128	-0.09	0.4661
VLDL4-C	0.17	0.1839	-0.09	0.4769
VLDL5-C	0.03	0.8008	-0.06	0.6178
VLDL-FC	0.24	0.0499	-0.15	0.2280
VLDL1-FC	0.38	0.0019	-0.17	0.1876
VLDL2-FC	0.27	0.0285	-0.11	0.3744
VLDL3-FC	0.23	0.0687	-0.12	0.3573
VLDL4-FC	0.15	0.2204	-0.14	0.2624
VLDL5-FC	-0.15	0.2296	-0.06	0.6312
VLDL-TG	0.32	0.0101	-0.13	0.3143
VLDL1-TG	0.38	0.0017	-0.14	0.2794
VLDL2-TG	0.27	0.0314	-0.15	0.2275
VLDL3-TG	0.20	0.1034	-0.11	0.3778
VLDL4-TG	0.16	0.1900	-0.05	0.6976
VLDL5-TG	0.01	0.9532	0.07	0.5984
VLDL-PL	0.28	0.0242	-0.14	0.2689
VLDL1-PL	0.40	0.0010	-0.12	0.3235
VLDL2-PL	0.30	0.0137	-0.14	0.2779
VLDL3-PL	0.23	0.0683	-0.12	0.3517
VLDL4-PL	0.21	0.0945	-0.08	0.5396
VLDL5-PL	0.08	0.5484	0.00	0.9716
VLDL-apoB	0.25	0.0470	-0.13	0.2927
**IDL**				
IDL-C	0.20	0.1154	-0.13	0.3047
IDL-FC	0.19	0.1198	-0.15	0.2327
IDL-TG	0.27	0.0283	-0.18	0.1525
IDL-PL	0.18	0.1542	-0.22	0.0799
IDL-apoB	0.14	0.2705	-0.11	0.3729
**LDL**				
LDL-C	0.08	0.5081	-0.08	0.5232
LDL1-C	-0.25	0.0454	-0.18	0.1412
LDL2-C	-0.24	0.0555	0.11	0.3762
LDL3-C	-0.09	0.4999	-0.06	0.6512
LDL4-C	0.32	0.0103	-0.13	0.2932
LDL5-C	0.41	0.0008	-0.12	0.3564
LDL6-C	0.26	0.0387	0.02	0.8522
LDL-FC	-0.05	0.7140	-0.11	0.3811
LDL1-FC	-0.26	0.0370	-0.19	0.1364
LDL2-FC	-0.31	0.0134	0.14	0.2770
LDL3-FC	-0.20	0.1097	-0.01	0.9072
LDL4-FC	0.23	0.0672	-0.13	0.2947
LDL5-FC	0.38	0.0019	-0.15	0.2364
LDL6-FC	0.26	0.0395	0.00	0.9686

LDL-TG	-0.01	0.9445	-0.11	0.3679
LDL1-TG	-0.19	0.1342	-0.09	0.4733
LDL2-TG	-0.30	0.0147	0.04	0.7611
LDL3-TG	-0.18	0.1449	-0.03	0.8017
LDL4-TG	0.18	0.1569	-0.13	0.3083
LDL5-TG	0.30	0.0170	-0.12	0.3216
LDL6-TG	0.16	0.1938	0.07	0.5573
LDL-PL	0.06	0.6612	-0.08	0.5065
LDL1-PL	-0.27	0.0311	-0.16	0.1910
LDL2-PL	-0.25	0.0464	0.10	0.4338
LDL3-PL	-0.11	0.4043	-0.05	0.6822
LDL4-PL	0.32	0.0090	-0.15	0.2263
LDL5-PL	0.41	0.0007	-0.12	0.3549
LDL6-PL	0.24	0.0556	0.02	0.8873
LDL-apoB	0.14	0.2805	-0.07	0.6018
LDL1-apoB	-0.28	0.0237	-0.15	0.2352
LDL2-apoB	-0.26	0.0333	0.11	0.3840
LDL3-apoB	-0.10	0.4219	-0.06	0.6086
LDL4-apoB	0.30	0.0139	-0.19	0.1351
LDL5-apoB	0.39	0.0012	-0.11	0.3912
LDL6-apoB	0.25	0.0484	0.01	0.9240
**HDL**				
HDL-C	-0.39	0.0013	0.00	0.9731
HDL1-C	-0.44	**0.0002**	0.06	0.6360
HDL2-C	-0.34	0.0060	0.07	0.5663
HDL3-C	-0.24	0.0552	-0.04	0.7320
HDL4-C	0.12	0.3497	0.03	0.8279
HDL-FC	-0.43	**0.0003**	-0.04	0.7527
HDL1-FC	-0.43	**0.0003**	-0.06	0.6141
HDL2-FC	-0.35	0.0042	-0.10	0.4477
HDL3-FC	-0.21	0.0889	-0.12	0.3278
HDL4-FC	0.07	0.5887	-0.01	0.9451
HDL-TG	-0.29	0.0179	-0.18	0.1477
HDL1-TG	-0.52	**<0.0001**	-0.12	0.3533
HDL2-TG	-0.28	0.0245	-0.14	0.2820
HDL3-TG	-0.11	0.4019	-0.14	0.2682
HDL4-TG	0.11	0.3669	-0.17	0.1673
HDL-PL	-0.46	**0.0001**	-0.06	0.6570
HDL1-PL	-0.46	**0.0001**	0.02	0.8441
HDL2-PL	-0.38	0.0020	0.02	0.8455
HDL3-PL	-0.27	0.0325	-0.07	0.5876
HDL4-PL	0.07	0.5552	-0.01	0.9541
HDL-apoA-I	-0.47	**0.0001**	-0.11	0.3749
HDL1-apoA-I	-0.49	**<0.0001**	0.00	0.9903
HDL2-apoA-I	-0.47	**0.0001**	0.02	0.8517
HDL3-apoA-I	-0.30	0.0147	-0.03	0.8072
HDL4-apoA-I	0.11	0.3875	-0.02	0.8852
HDL-apoA-II	-0.06	0.6506	-0.18	0.1449
HDL1-apoA-II	-0.44	**0.0002**	-0.07	0.6012
HDL2-apoA-II	-0.35	0.0040	-0.07	0.5655
HDL3-apoA-II	-0.04	0.7380	-0.15	0.2421
HDL4-apoA-II	0.21	0.0865	-0.06	0.6145

Spearman correlation analyses were used to evaluate the associations between the serum levels of lipoproteins and waist circumference. *p*-values < 0.0005 are considered statistically significant after a Bonferroni correction for multiple testing and are depicted in bold. ApoA-I, apolipoprotein A-I; apoA-II, apolipoprotein A-II; apoB,

apolipoprotein B; BMI, body mass index; C, cholesterol; FC, free cholesterol; HV, healthy volunteer; HDL, high-density lipoprotein; IDL, intermediate-density lipoprotein; IL-6, interleukin-6; LDL, low-density lipoprotein; MS, metabolic syndrome patient; PL, phospholipid; r, Spearman's correlation coefficient; TG, triglyceride; VLDL, very low-density lipoprotein.
