# Supplementary material for: Serum Levels of Adiponectin Are Strongly Associated with Lipoprotein Subclasses in Healthy Volunteers but Not in Patients with Metabolic Syndrome
Source: Int J Mol Sci. 2024 May 6;25(9):5050. doi: 10.3390/ijms25095050 (PMC11084877; doi:10.3390/ijms25095050)
Supplement: Supplementary file 1 [file ijms-25-05050-s001.zip › Table S11.pdf]

Table S11. Correlation analyses of serum levels of lipoprotein classes and subclasses with IL-6, performed separately for the HVs and patients with MS.

	**IL-6 (pg/mL)**			
	**HV (N=65)**		**MS (N=65)**	
**Variable (mg/dL)**	**r**	**p-value**	**r**	**p-value**
**VLDL**				
VLDL-C	0.20	0.1019	-0.38	0.0018
VLDL1-C	0.22	0.0808	-0.41	0.0007
VLDL2-C	0.23	0.0663	-0.36	0.0031
VLDL3-C	0.23	0.0636	-0.33	0.0071
VLDL4-C	0.21	0.1006	-0.27	0.0291
VLDL5-C	0.03	0.8311	-0.02	0.8530
VLDL-FC	0.17	0.1717	-0.37	0.0021
VLDL1-FC	0.24	0.0503	-0.42	0.0006
VLDL2-FC	0.21	0.0952	-0.37	0.0027
VLDL3-FC	0.19	0.1377	-0.36	0.0032
VLDL4-FC	0.20	0.1082	-0.32	0.0093
VLDL5-FC	-0.18	0.1446	-0.20	0.1152
VLDL-TG	0.19	0.1289	-0.38	0.0016
VLDL1-TG	0.19	0.1358	-0.40	0.0010
VLDL2-TG	0.23	0.0649	-0.36	0.0031
VLDL3-TG	0.18	0.1592	-0.35	0.0047
VLDL4-TG	0.11	0.3687	-0.30	0.0157
VLDL5-TG	-0.09	0.4739	-0.06	0.6552
VLDL-PL	0.18	0.1620	-0.37	0.0024
VLDL1-PL	0.24	0.0589	-0.39	0.0013
VLDL2-PL	0.25	0.0408	-0.35	0.0038
VLDL3-PL	0.18	0.1575	-0.36	0.0030
VLDL4-PL	0.18	0.1509	-0.29	0.0185
VLDL5-PL	0.03	0.8088	-0.06	0.6109
VLDL-apoB	0.14	0.2655	-0.36	0.0032
**IDL**				
IDL-C	0.26	0.0356	-0.26	0.0343
IDL-FC	0.26	0.0360	-0.30	0.0141
IDL-TG	0.17	0.1846	-0.42	**0.0004**
IDL-PL	0.27	0.0303	-0.38	0.0016
IDL-apoB	0.18	0.1547	-0.23	0.0668
**LDL**				
LDL-C	0.16	0.2053	0.11	0.3633
LDL1-C	0.06	0.6503	0.09	0.4983
LDL2-C	-0.09	0.4579	0.37	0.0028
LDL3-C	0.06	0.6493	0.20	0.1139
LDL4-C	0.23	0.0695	0.04	0.7768
LDL5-C	0.17	0.1835	0.00	0.9846
LDL6-C	0.16	0.2033	-0.09	0.4649
LDL-FC	0.12	0.3327	0.14	0.2683
LDL1-FC	0.09	0.4919	0.07	0.5893
LDL2-FC	-0.11	0.4035	0.34	0.0054
LDL3-FC	0.03	0.8315	0.22	0.0722
LDL4-FC	0.21	0.1005	0.08	0.5300
LDL5-FC	0.17	0.1814	0.04	0.7247
LDL6-FC	0.17	0.1800	-0.04	0.7375

LDL-TG	0.15	0.2328	-0.07	0.6052
LDL1-TG	0.07	0.5964	-0.07	0.5716
LDL2-TG	0.02	0.8542	0.19	0.1205
LDL3-TG	-0.11	0.3743	0.33	0.0074
LDL4-TG	0.17	0.1658	0.12	0.3426
LDL5-TG	0.17	0.1754	-0.12	0.3397
LDL6-TG	0.04	0.7527	-0.02	0.8670
LDL-PL	0.17	0.1877	0.16	0.2140
LDL1-PL	0.03	0.7907	0.13	0.2920
LDL2-PL	-0.08	0.5516	0.37	0.0024
LDL3-PL	0.06	0.6449	0.22	0.0852
LDL4-PL	0.24	0.0556	0.03	0.7947
LDL5-PL	0.17	0.1650	0.01	0.9529
LDL6-PL	0.12	0.3248	-0.09	0.4878
LDL-apoB	0.18	0.1576	0.05	0.6803
LDL1-apoB	0.02	0.8484	0.11	0.3663
LDL2-apoB	-0.11	0.3849	0.36	0.0029
LDL3-apoB	0.06	0.6426	0.23	0.0706
LDL4-apoB	0.24	0.0545	-0.01	0.9656
LDL5-apoB	0.18	0.1529	0.02	0.8969
LDL6-apoB	0.15	0.2292	-0.12	0.3597
**HDL**				
HDL-C	-0.16	0.2089	0.14	0.2540
HDL1-C	-0.14	0.2571	0.14	0.2704
HDL2-C	-0.10	0.4471	0.17	0.1720
HDL3-C	-0.04	0.7688	0.05	0.6672
HDL4-C	0.06	0.6318	0.00	0.9916
HDL-FC	-0.13	0.3042	0.14	0.2781
HDL1-FC	-0.15	0.2370	0.11	0.3923
HDL2-FC	-0.11	0.3925	0.13	0.2991
HDL3-FC	-0.02	0.8921	0.07	0.5558
HDL4-FC	0.11	0.3772	0.03	0.7859
HDL-TG	0.00	0.9763	-0.15	0.2250
HDL1-TG	-0.13	0.3192	-0.04	0.7280
HDL2-TG	-0.04	0.7789	-0.05	0.6771
HDL3-TG	0.07	0.5777	-0.16	0.2092
HDL4-TG	0.10	0.4339	-0.38	0.0017
HDL-PL	-0.16	0.1973	0.13	0.2966
HDL1-PL	-0.14	0.2625	0.16	0.2102
HDL2-PL	-0.15	0.2366	0.14	0.2722
HDL3-PL	-0.10	0.4371	0.06	0.6077
HDL4-PL	0.09	0.4597	-0.03	0.8060
HDL-apoA-I	-0.16	0.1906	-0.05	0.7072
HDL1-apoA-I	-0.18	0.1512	0.12	0.3506
HDL2-apoA-I	-0.27	0.0281	0.07	0.6054
HDL3-apoA-I	-0.07	0.5596	-0.03	0.8355
HDL4-apoA-I	0.11	0.3891	-0.11	0.3878
HDL-apoA-II	0.02	0.8487	-0.25	0.0429
HDL1-apoA-II	-0.24	0.0567	-0.03	0.7851
HDL2-apoA-II	-0.24	0.0514	-0.08	0.5424
HDL3-apoA-II	0.01	0.9544	-0.16	0.2060
HDL4-apoA-II	0.12	0.3288	-0.20	0.1018

Spearman correlation analyses were used to evaluate the associations between the serum levels of lipoproteins and IL-6. *p*-values < 0.0005 are considered statistically significant after a Bonferroni correction for multiple testing and are depicted in bold. ApoA-I, apolipoprotein A-I; apoA-II, apolipoprotein A-II; apoB, apolipoprotein B; C,

cholesterol; FC, free cholesterol; HV, healthy volunteer; HDL, high-density lipoprotein; IDL, intermediate-density lipoprotein; IL-6, interleukin-6; LDL, low-density lipoprotein; MS, metabolic syndrome patient; PL, phospholipid; r, Spearman's correlation coefficient; TG, triglyceride; VLDL, very low-density lipoprotein.
