# Supplementary material for: Serum Levels of Adiponectin Are Strongly Associated with Lipoprotein Subclasses in Healthy Volunteers but Not in Patients with Metabolic Syndrome
Source: Int J Mol Sci. 2024 May 6;25(9):5050. doi: 10.3390/ijms25095050 (PMC11084877; doi:10.3390/ijms25095050)
Supplement: Supplementary file 1 [file ijms-25-05050-s001.zip › Table S12.pdf]

Table S12. Correlation analyses of serum levels of lipoprotein classes and subclasses with CRP, performed separately for the HVs and patients with MS.

	CRP (μg/mL)			
	HV (N=65)		MS (N=65)	
**Variable (mg/dL)**	**r**	**p-value**	**r**	**p-value**
**VLDL**				
VLDL-C	0.13	0.2884	-0.12	0.3595
VLDL1-C	0.10	0.4271	-0.14	0.2751
VLDL2-C	0.15	0.2350	-0.12	0.3536
VLDL3-C	0.16	0.2175	-0.09	0.4789
VLDL4-C	0.16	0.1959	-0.06	0.6257
VLDL5-C	0.00	0.9782	-0.01	0.9331
VLDL-FC	0.09	0.4820	-0.15	0.2342
VLDL1-FC	0.15	0.2473	-0.14	0.2535
VLDL2-FC	0.12	0.3350	-0.09	0.4798
VLDL3-FC	0.11	0.3872	-0.09	0.4742
VLDL4-FC	0.17	0.1818	-0.10	0.4457
VLDL5-FC	-0.05	0.6638	0.04	0.7720
VLDL-TG	0.08	0.5063	-0.12	0.3378
VLDL1-TG	0.04	0.7241	-0.16	0.2161
VLDL2-TG	0.11	0.3681	-0.12	0.3262
VLDL3-TG	0.08	0.5482	-0.10	0.4345
VLDL4-TG	0.07	0.5970	-0.09	0.4856
VLDL5-TG	-0.05	0.7058	0.01	0.9216
VLDL-PL	0.09	0.4914	-0.12	0.3593
VLDL1-PL	0.10	0.4268	-0.10	0.4107
VLDL2-PL	0.15	0.2340	-0.09	0.4949
VLDL3-PL	0.12	0.3420	-0.08	0.5142
VLDL4-PL	0.15	0.2313	-0.07	0.5969
VLDL5-PL	0.01	0.9257	0.03	0.8428
VLDL-apoB	0.08	0.5225	-0.08	0.5354
**IDL**				
IDL-C	0.22	0.0848	-0.03	0.7926
IDL-FC	0.21	0.0979	-0.08	0.5422
IDL-TG	0.05	0.6712	-0.14	0.2622
IDL-PL	0.14	0.2664	-0.16	0.2113
IDL-apoB	0.18	0.1430	0.01	0.9659
**LDL**				
LDL-C	0.12	0.3284	0.00	0.9752
LDL1-C	0.03	0.8105	-0.01	0.9326
LDL2-C	-0.02	0.8866	0.19	0.1291
LDL3-C	0.09	0.4981	0.02	0.8903
LDL4-C	0.19	0.1314	-0.09	0.4818
LDL5-C	0.14	0.2731	-0.09	0.4930
LDL6-C	0.10	0.4498	-0.02	0.8468
LDL-FC	0.05	0.7180	-0.02	0.8526
LDL1-FC	0.05	0.6777	-0.02	0.8997
LDL2-FC	-0.06	0.6302	0.17	0.1668
LDL3-FC	-0.02	0.8995	0.00	0.9862
LDL4-FC	0.14	0.2684	-0.08	0.5419
LDL5-FC	0.12	0.3401	-0.08	0.5204
LDL6-FC	0.11	0.3844	-0.04	0.7397

LDL-TG	0.11	0.3757	0.11	0.3623
LDL1-TG	0.08	0.5186	0.08	0.5381
LDL2-TG	0.05	0.7004	0.19	0.1262
LDL3-TG	0.01	0.9306	0.19	0.1294
LDL4-TG	0.17	0.1858	0.12	0.3604
LDL5-TG	0.11	0.3822	0.08	0.5490
LDL6-TG	0.04	0.7510	0.12	0.3242
LDL-PL	0.14	0.2699	0.03	0.7850
LDL1-PL	0.05	0.6707	0.02	0.8611
LDL2-PL	0.00	0.9977	0.19	0.1354
LDL3-PL	0.09	0.4944	0.04	0.7679
LDL4-PL	0.20	0.1023	-0.09	0.4561
LDL5-PL	0.14	0.2788	-0.07	0.5756
LDL6-PL	0.08	0.5186	-0.03	0.8181
LDL-apoB	0.15	0.2298	0.04	0.7233
LDL1-apoB	0.06	0.6443	0.03	0.7826
LDL2-apoB	0.00	0.9783	0.24	0.0591
LDL3-apoB	0.09	0.4590	0.06	0.6336
LDL4-apoB	0.20	0.1077	-0.09	0.4578
LDL5-apoB	0.14	0.2774	-0.03	0.8004
LDL6-apoB	0.09	0.4516	0.01	0.9394
**HDL**				
HDL-C	-0.16	0.1911	-0.14	0.2624
HDL1-C	-0.18	0.1426	0.04	0.7313
HDL2-C	-0.06	0.6330	0.05	0.6938
HDL3-C	-0.05	0.7139	-0.09	0.4601
HDL4-C	-0.04	0.7532	-0.27	0.0298
HDL-FC	-0.18	0.1466	-0.10	0.4121
HDL1-FC	-0.15	0.2412	-0.08	0.5207
HDL2-FC	-0.02	0.8860	-0.02	0.8571
HDL3-FC	-0.01	0.9262	-0.12	0.3374
HDL4-FC	0.01	0.9667	-0.21	0.0998
HDL-TG	-0.05	0.7171	-0.03	0.8087
HDL1-TG	-0.16	0.2080	0.02	0.9010
HDL2-TG	-0.02	0.8832	0.10	0.4228
HDL3-TG	0.04	0.7328	0.08	0.5129
HDL4-TG	-0.01	0.9186	-0.15	0.2257
HDL-PL	-0.12	0.3543	-0.04	0.7348
HDL1-PL	-0.15	0.2281	0.10	0.4506
HDL2-PL	-0.05	0.7217	0.07	0.5588
HDL3-PL	-0.01	0.9161	-0.05	0.6977
HDL4-PL	0.04	0.7387	-0.21	0.0980
HDL-apoA-I	-0.18	0.1516	-0.23	0.0619
HDL1-apoA-I	-0.18	0.1572	0.04	0.7736
HDL2-apoA-I	-0.16	0.2043	-0.02	0.8964
HDL3-apoA-I	-0.03	0.8146	-0.08	0.5310
HDL4-apoA-I	-0.02	0.8469	-0.31	0.0107
HDL-apoA-II	0.04	0.7655	-0.33	0.0081
HDL1-apoA-II	-0.17	0.1742	0.03	0.8257
HDL2-apoA-II	-0.07	0.5605	0.03	0.8204
HDL3-apoA-II	0.07	0.5929	-0.10	0.4320
HDL4-apoA-II	0.02	0.9026	-0.37	0.0025

Spearman correlation analyses were used to evaluate the associations between the serum levels of lipoproteins and CRP. *p*-values < 0.0005 are considered statistically significant after a Bonferroni correction for multiple testing. ApoA-I, apolipoprotein A-I; apoA-II, apolipoprotein A-II; apoB, apolipoprotein B; C, cholesterol; CRP, C-reactive

protein; FC, free cholesterol; HV, healthy volunteer; HDL, high-density lipoprotein; IDL, intermediate-density lipoprotein; LDL, low-density lipoprotein; MS, metabolic syndrome patient; PL, phospholipid; r, Spearman's correlation coefficient; TG, triglyceride; VLDL, very low-density lipoprotein.
