# Supplementary material for: Serum Levels of Adiponectin Are Strongly Associated with Lipoprotein Subclasses in Healthy Volunteers but Not in Patients with Metabolic Syndrome
Source: Int J Mol Sci. 2024 May 6;25(9):5050. doi: 10.3390/ijms25095050 (PMC11084877; doi:10.3390/ijms25095050)
Supplement: Supplementary file 1 [file ijms-25-05050-s001.zip › Table S13.pdf]

Table S13. Correlation analyses of serum levels of lipoprotein classes and subclasses with MetSSS, performed separately for the HVs and patients with MS.

	**MetSSS**			
	**HV (N=65)**		**MS (N=65)**	
**Variable (mg/dL)**	**r**	**p-value**	**r**	**p-value**
**VLDL**				
VLDL-C	0.31	0.0111	0.14	0.2557
VLDL1-C	0.36	0.0029	0.16	0.1977
VLDL2-C	0.28	0.0261	0.12	0.3432
VLDL3-C	0.26	0.0344	0.14	0.2632
VLDL4-C	0.20	0.1055	0.09	0.4947
VLDL5-C	-0.09	0.4657	-0.13	0.3109
VLDL-FC	0.31	0.0134	0.14	0.2829
VLDL1-FC	0.45	**0.0001**	0.16	0.2158
VLDL2-FC	0.33	0.0077	0.17	0.1810
VLDL3-FC	0.27	0.0270	0.15	0.2192
VLDL4-FC	0.18	0.1444	0.04	0.7355
VLDL5-FC	-0.19	0.1256	0.08	0.5444
VLDL-TG	0.42	0.0005	0.18	0.1446
VLDL1-TG	0.51	**<0.0001**	0.16	0.1998
VLDL2-TG	0.34	0.0055	0.14	0.2699
VLDL3-TG	0.27	0.0272	0.16	0.2058
VLDL4-TG	0.22	0.0806	0.15	0.2238
VLDL5-TG	-0.07	0.6069	0.13	0.3183
VLDL-PL	0.35	0.0042	0.15	0.2228
VLDL1-PL	0.49	**<0.000**	0.18	0.1415
VLDL2-PL	0.37	0.0022	0.15	0.2344
VLDL3-PL	0.29	0.0174	0.16	0.2042
VLDL4-PL	0.25	0.0439	0.10	0.4263
VLDL5-PL	0.01	0.9460	0.02	0.8556
VLDL-apoB	0.30	0.0145	0.13	0.3201
**IDL**				
IDL-C	0.28	0.0235	0.07	0.5936
IDL-FC	0.28	0.0259	0.05	0.6641
IDL-TG	0.35	0.0038	0.15	0.2327
IDL-PL	0.29	0.0171	0.06	0.6375
IDL-apoB	0.25	0.0427	0.04	0.7639
**LDL**				
LDL-C	0.16	0.2069	-0.16	0.1911
LDL1-C	-0.29	0.0174	-0.26	0.0329
LDL2-C	-0.26	0.0383	-0.10	0.4257
LDL3-C	-0.03	0.7824	-0.20	0.1096
LDL4-C	0.41	0.0007	-0.20	0.1061
LDL5-C	0.54	**<0.0000**	-0.15	0.2275
LDL6-C	0.43	0.0004	0.16	0.1891
LDL-FC	0.03	0.7978	-0.24	0.0580
LDL1-FC	-0.31	0.0134	-0.27	0.0307
LDL2-FC	-0.33	0.0078	-0.08	0.5264
LDL3-FC	-0.18	0.1436	-0.16	0.1946
LDL4-FC	0.30	0.0147	-0.22	0.0794
LDL5-FC	0.48	**0.0001**	-0.20	0.1170
LDL6-FC	0.36	0.0036	0.12	0.3281

LDL-TG	0.10	0.4183	-0.05	0.6851
LDL1-TG	-0.18	0.1595	-0.04	0.7358
LDL2-TG	-0.26	0.0342	-0.04	0.7421
LDL3-TG	-0.17	0.1858	-0.24	0.0543
LDL4-TG	0.25	0.0411	-0.21	0.0950
LDL5-TG	0.41	0.0006	-0.03	0.8229
LDL6-TG	0.30	0.0146	0.21	0.0919
LDL-PL	0.11	0.3620	-0.20	0.1105
LDL1-PL	-0.32	0.0091	-0.28	0.0252
LDL2-PL	-0.28	0.0215	-0.13	0.3178
LDL3-PL	-0.06	0.6202	-0.21	0.0901
LDL4-PL	0.41	0.0006	-0.22	0.0819
LDL5-PL	0.54	**<0.0001**	-0.17	0.1864
LDL6-PL	0.40	0.0011	0.16	0.1996
LDL-apoB	0.25	0.0479	-0.07	0.5970
LDL1-apoB	-0.32	0.0083	-0.24	0.0505
LDL2-apoB	-0.29	0.0210	-0.10	0.4403
LDL3-apoB	-0.04	0.7379	-0.24	0.0584
LDL4-apoB	0.42	0.0006	-0.24	0.0532
LDL5-apoB	0.53	**<0.0001**	-0.14	0.2680
LDL6-apoB	0.43	**0.0003**	0.18	0.1420
**HDL**				
HDL-C	-0.43	**0.0003**	-0.18	0.1459
HDL1-C	-0.44	**0.0003**	0.02	0.8768
HDL2-C	-0.41	0.0007	0.00	0.9960
HDL3-C	-0.31	0.0110	-0.08	0.5371
HDL4-C	0.14	0.2800	-0.10	0.4172
HDL-FC	-0.48	**0.0001**	-0.16	0.2005
HDL1-FC	-0.41	0.0006	-0.13	0.3062
HDL2-FC	-0.39	0.0013	-0.17	0.1778
HDL3-FC	-0.22	0.0806	-0.19	0.1197
HDL4-FC	0.06	0.6529	-0.11	0.3755
HDL-TG	-0.32	0.0086	-0.06	0.6511
HDL1-TG	-0.52	**<0.0001**	-0.06	0.6570
HDL2-TG	-0.32	0.0100	-0.04	0.7628
HDL3-TG	-0.13	0.3146	0.01	0.9452
HDL4-TG	0.16	0.2081	0.02	0.8768
HDL-PL	-0.52	**<0.0001**	-0.19	0.1362
HDL1-PL	-0.46	**0.0001**	-0.02	0.8549
HDL2-PL	-0.44	**0.0002**	-0.02	0.8987
HDL3-PL	-0.35	0.0047	-0.11	0.3633
HDL4-PL	0.04	0.7552	-0.10	0.4127
HDL-apoA-I	-0.48	**<0.0001**	-0.19	0.1236
HDL1-apoA-I	-0.47	**0.0001**	-0.05	0.6686
HDL2-apoA-I	-0.47	**0.0001**	0.00	0.9938
HDL3-apoA-I	-0.34	0.0062	0.02	0.8488
HDL4-apoA-I	0.13	0.3146	-0.09	0.4522
HDL-apoA-II	0.01	0.9530	-0.06	0.6567
HDL1-apoA-II	-0.36	0.0033	0.03	0.8053
HDL2-apoA-II	-0.28	0.0227	0.06	0.6280
HDL3-apoA-II	0.00	0.9914	0.00	0.9836
HDL4-apoA-II	0.27	0.0327	-0.06	0.6568

Spearman correlation analyses were used to evaluate the associations between the serum levels of lipoproteins and MetSSS. *p*-values < 0.0005 are considered statistically significant after a Bonferroni correction for multiple testing. ApoA-I, apolipoprotein A-I; apoA-II, apolipoprotein A-II; apoB, apolipoprotein B; C, cholesterol; FC, free

cholesterol; HV, healthy volunteer; HDL, high-density lipoprotein; IDL, intermediate-density lipoprotein; LDL, low-density lipoprotein; MetSSS, metabolic syndrome severity score; MS, metabolic syndrome patient; PL, phospholipid; r, Spearman's correlation coefficient; TG, triglyceride; VLDL, very low-density lipoprotein.
