# Supplementary material for: Serum Levels of Adiponectin Are Strongly Associated with Lipoprotein Subclasses in Healthy Volunteers but Not in Patients with Metabolic Syndrome
Source: Int J Mol Sci. 2024 May 6;25(9):5050. doi: 10.3390/ijms25095050 (PMC11084877; doi:10.3390/ijms25095050)
Supplement: Supplementary file 1 [file ijms-25-05050-s001.zip › Table S2.pdf]

Table S2. Differences in serum levels of lipoproteins between HVs and patients with MS.

Variable (mg/dL)	All (N=130)	HV (N=65)	MS (N=65)	p
**VLDL**				
VLDL-C	20.5 (12.1, 32.2)	16.2 (11.2, 24.1)	24.2 (14.1, 39.1)	0.0005
VLDL1-C	6.3 (4.0, 12.3)	5.4 (3.1, 8.1)	9.2 (5.0, 15.9)	**< 0.0001**
VLDL2-C	2.7 (1.6, 4.8)	2.3 (1.4, 3.5)	3.2 (1.7, 6.1)	0.0151
VLDL3-C	3.7 (2.0, 5.6)	2.8 (1.9, 4.5)	4.6 (2.2, 7.5)	0.0015
VLDL4-C	5.2 (3.7, 7.4)	4.5 (3.4, 6.1)	6.5 (4.3, 8.8)	0.0008
VLDL5-C	1.2 (0.8, 1.6)	1.1 (0.8, 1.5)	1.2 (0.8, 1.6)	0.9166
VLDL-FC	9.0 (6.1, 13.4)	7.6 (5.6, 10.6)	10.8 (6.6, 16.0)	**0.0003**
VLDL1-FC	2.3 (1.1, 4.1)	1.8 (0.9, 2.9)	3.2 (1.4, 5.0)	**0.0002**
VLDL2-FC	1.0 (0.5, 2.2)	0.9 (0.4, 1.5)	1.4 (0.7, 2.6)	0.0017
VLDL3-FC	1.5 (0.8, 2.6)	1.1 (0.7, 2.0)	2.1 (0.9, 3.3)	**0.0003**
VLDL4-FC	2.4 (1.5, 3.6)	2.0 (1.3, 2.7)	3.0 (1.8, 4.3)	**0.0003**
VLDL5-FC	0.6 (0.4, 1.0)	0.6 (0.4, 0.9)	0.8 (0.5, 1.0)	0.0533
VLDL-TG	69.3 (43.8, 116.4)	54.5 (39.0, 80.3)	92.3 (58.8, 131.9)	**< 0.0001**
VLDL1-TG	29.2 (18.7, 55.4)	25.1 (13.8, 39.4)	47.5 (26.2, 72.8)	**< 0.0001**
VLDL2-TG	10.3 (6.1, 18.1)	8.9 (5.0, 11.6)	13.3 (8.2, 21.1)	**< 0.0001**
VLDL3-TG	9.7 (5.6, 14.9)	7.6 (4.8, 11.9)	12.2 (6.6, 19.2)	**0.0003**
VLDL4-TG	8.8 (5.9, 11.6)	6.8 (5.2, 9.2)	10.3 (8.0, 13.6)	**< 0.0001**
VLDL5-TG	2.7 (2.4, 3.3)	2.6 (2.2, 2.9)	3.0 (2.6, 3.6)	**0.0001**
VLDL-PL	18.8 (12.4, 28.4)	15.6 (10.7, 21.5)	22.8 (14.3, 33.1)	**< 0.0001**
VLDL1-PL	4.8 (2.9, 9.3)	4.0 (2.3, 6.5)	7.5 (3.9, 11.4)	**< 0.0001**
VLDL2-PL	2.7 (1.6, 4.7)	2.2 (1.3, 3.1)	3.3 (1.9, 5.5)	**0.0003**
VLDL3-PL	3.5 (1.9, 5.2)	2.7 (1.7, 4.3)	4.4 (2.4, 6.7)	**0.0003**
VLDL4-PL	4.5 (3.1, 6.0)	3.8 (2.8, 4.9)	5.2 (3.9, 7.0)	**< 0.0001**
VLDL5-PL	1.6 (1.2, 2.0)	1.5 (1.1, 2.0)	1.8 (1.2, 2.1)	0.0539
VLDL-apoB	7.9 (5.3, 11.0)	6.2 (4.1, 8.8)	9.2 (6.6, 13.4)	**< 0.0001**
**IDL**				
IDL-C	15.9 (11.1, 21.8)	14.5 (10.0, 18.9)	18.3 (13.3, 26.6)	0.0006
IDL-FC	4.6 (3.1, 6.2)	4.0 (2.9, 5.4)	5.3 (3.8, 7.8)	0.0013
IDL-TG	9.4 (5.5, 18.2)	7.1 (4.1, 11.6)	13.8 (7.3, 22.5)	**< 0.0001**
IDL-PL	7.8 (6.2, 11.1)	7.3 (6.1, 9.4)	9.4 (6.5, 12.7)	0.0199
IDL-apoB	6.0 (4.5, 7.8)	5.4 (4.0, 6.9)	6.9 (5.1, 9.0)	**< 0.0001**
**LDL**				
LDL-C	125.1 (113.0, 153.1)	137.4 (121.8, 154.3)	117.1 (90.4, 148.1)	0.0006
LDL1-C	28.1 (23.4, 34.6)	30.4 (26.5, 36.7)	24.5 (21.3, 30.1)	**< 0.0001**
LDL2-C	21.6 (14.8, 26.6)	25.9 (20.1, 30.7)	17.4 (12.2, 22.3)	**< 0.0001**
LDL3-C	21.4 (15.4, 27.6)	23.8 (21.0, 29.1)	17.6 (9.9, 22.2)	**< 0.0001**
LDL4-C	19.2 (14.2, 24.2)	20.3 (15.8, 24.4)	17.9 (11.2, 24.1)	0.0519
LDL5-C	16.5 (12.7, 22.3)	15.9 (12.4, 21.9)	17.4 (13.1, 23.8)	0.2434
LDL6-C	20.1 (16.6, 25.8)	19.2 (16.5, 22.8)	22.1 (17.1, 28.8)	0.0387
LDL-FC	38.7 (35.2, 46.3)	42.2 (38.0, 47.3)	37.3 (28.8, 45.1)	0.0011
LDL1-FC	9.0 (7.7, 10.9)	9.5 (8.4, 11.3)	8.2 (7.1, 9.8)	0.0006
LDL2-FC	7.5 (5.3, 8.9)	8.6 (6.7, 9.8)	6.4 (4.9, 7.7)	**< 0.0001**
LDL3-FC	7.2 (5.5, 8.6)	7.9 (7.1, 9.2)	6.5 (4.3, 7.3)	**< 0.0001**
LDL4-FC	6.2 (4.9, 7.4)	6.6 (5.3, 7.6)	5.8 (4.5, 7.1)	0.0463
LDL5-FC	5.3 (4.4, 6.7)	5.3 (4.2, 6.5)	5.3 (4.6, 6.8)	0.2549
LDL6-FC	6.1 (5.1, 7.1)	5.8 (4.9, 6.5)	6.4 (5.3, 7.6)	0.0226
LDL-TG	21.7 (18.1, 25.9)	20.0 (16.5, 23.6)	23.8 (20.5, 28.3)	**< 0.0001**

Variable (mg/dL)	All (N=130)	HV (N=65)	MS (N=65)	p
LDL1-TG	6.8 (5.7, 8.2)	6.4 (5.1, 7.6)	7.5 (6.3, 8.6)	0.0008
LDL2-TG	2.6 (2.1, 3.2)	2.7 (2.1, 3.2)	2.6 (2.1, 3.2)	0.5220
LDL3-TG	2.5 (1.9, 3.0)	2.5 (2.1, 3.0)	2.4 (1.9, 3.0)	0.3663
LDL4-TG	2.7 (1.9, 3.4)	2.5 (1.6, 3.0)	2.9 (2.1, 3.6)	0.0026
LDL5-TG	2.5 (1.8, 3.6)	2.0 (1.4, 3.0)	3.1 (2.2, 4.3)	< **0.0001**
LDL6-TG	3.2 (2.6, 4.1)	2.8 (2.3, 3.4)	3.9 (2.9, 4.7)	< **0.0001**
LDL-PL	70.9 (63.5, 83.3)	75.9 (67.9, 85.4)	66.6 (51.5, 80.2)	0.0015
LDL1-PL	15.5 (13.3, 18.7)	16.6 (14.7, 19.7)	14.3 (12.1, 16.9)	0.0005
LDL2-PL	12.1 (8.4, 14.5)	14.0 (11.1, 16.2)	9.6 (7.0, 12.4)	< **0.0001**
LDL3-PL	11.9 (8.9, 15.0)	13.2 (11.3, 15.6)	10.1 (6.3, 12.4)	< **0.0001**
LDL4-PL	10.4 (8.0, 13.0)	11.4 (9.0, 13.1)	10.2 (6.7, 12.9)	0.1051
LDL5-PL	9.2 (7.3, 12.0)	8.7 (7.0, 11.8)	9.6 (7.5, 12.6)	0.1638
LDL6-PL	11.6 (10.1, 14.3)	11.2 (10.0, 12.8)	12.6 (10.2, 15.3)	0.0291
LDL-apoB	81.7 (70.0, 94.6)	82.3 (72.8, 94.9)	78.5 (65.0, 92.1)	0.2648
LDL1-apoB	14.7 (12.7, 17.8)	15.3 (13.4, 18.4)	13.9 (11.5, 16.3)	0.0046
LDL2-apoB	12.3 (8.9, 14.7)	13.8 (11.2, 16.4)	9.9 (7.3, 12.8)	< **0.0001**
LDL3-apoB	12.5 (9.8, 15.6)	13.7 (12.0, 16.4)	10.8 (6.9, 13.2)	< **0.0001**
LDL4-apoB	12.4 (9.4, 15.7)	13.4 (9.7, 15.3)	12.3 (8.4, 16.2)	0.4409
LDL5-apoB	11.9 (9.2, 16.2)	11.3 (8.4, 15.2)	12.8 (9.9, 17.7)	0.0306
LDL6-apoB	16.2 (13.6, 22.0)	15.8 (13.4, 18.0)	18.0 (14.1, 23.9)	0.0138
**HDL**				
HDL-C	58.6 (51.3, 69.2)	65.2 (57.7, 74.5)	52.7 (47.9, 60.6)	< **0.0001**
HDL1-C	17.2 (13.8, 22.5)	18.4 (15.1, 26.8)	15.7 (12.9, 20.1)	0.0011
HDL2-C	8.6 (7.5, 10.2)	9.5 (8.3, 12.6)	8.2 (7.3, 9.7)	0.0012
HDL3-C	11.2 (10.0, 13.2)	12.2 (10.7, 13.6)	10.4 (9.6, 11.9)	**0.0001**
HDL4-C	20.5 (17.2, 23.9)	22.3 (18.2, 24.7)	19.0 (16.0, 22.7)	0.0013
HDL-FC	14.5 (12.5, 16.7)	16.0 (14.1, 18.9)	13.5 (11.8, 15.0)	< **0.0001**
HDL1-FC	5.0 (4.1, 6.1)	5.4 (4.8, 7.4)	4.3 (3.7, 5.2)	< **0.0001**
HDL2-FC	2.5 (2.1, 2.9)	2.7 (2.4, 3.1)	2.3 (2.0, 2.7)	0.0015
HDL3-FC	2.8 (2.3, 3.2)	3.0 (2.6, 3.4)	2.5 (2.1, 3.0)	0.0012
HDL4-FC	4.6 (3.9, 5.4)	4.9 (4.0, 5.7)	4.4 (3.8, 5.2)	0.0326
HDL-TG	10.5 (9.0, 13.3)	9.9 (8.7, 11.8)	11.4 (9.7, 13.6)	0.0058
HDL1-TG	3.3 (2.6, 4.4)	3.0 (2.5, 4.2)	3.6 (2.7, 4.6)	0.3258
HDL2-TG	1.8 (1.5, 2.3)	1.6 (1.3, 2.1)	2.0 (1.6, 2.5)	0.0013
HDL3-TG	2.3 (1.9, 2.8)	2.1 (1.7, 2.5)	2.7 (2.1, 3.1)	**0.0002**
HDL4-TG	3.6 (3.0, 4.3)	3.4 (2.5, 3.9)	3.7 (3.3, 4.7)	**0.0003**
HDL-PL	81.9 (72.1, 93.2)	89.4 (79.2, 99.8)	77.1 (67.5, 84.5)	< **0.0001**
HDL1-PL	20.5 (16.8, 26.5)	22.1 (18.6, 33.9)	18.9 (14.8, 22.9)	0.0021
HDL2-PL	13.8 (11.7, 15.9)	14.4 (12.6, 18.6)	13.2 (10.9, 15.4)	0.0175
HDL3-PL	18.2 (15.9, 20.6)	19.1 (17.4, 20.9)	17.1 (15.2, 19.6)	0.0037
HDL4-PL	28.8 (25.4, 31.8)	29.9 (26.4, 32.7)	26.3 (23.1, 30.7)	0.0033
HDL-apoA-I	159.3 (144.5, 178.2)	167.8 (155.7, 183.7)	149.2 (138.1, 166.2)	< **0.0001**
HDL1-apoA-I	26.3 (20.0, 34.7)	27.5 (22.3, 45.4)	24.9 (18.5, 30.1)	0.0067
HDL2-apoA-I	18.7 (16.3, 22.1)	19.6 (17.2, 23.3)	17.5 (15.2, 20.6)	0.0036
HDL3-apoA-I	30.2 (26.7, 33.3)	30.8 (27.5, 33.5)	28.8 (24.9, 32.5)	0.0396
HDL4-apoA-I	79.5 (70.8, 89.0)	81.9 (72.7, 91.1)	75.5 (68.3, 86.5)	0.0231
HDL-apoA-II	35.2 (32.2, 38.4)	36.0 (33.6, 38.6)	33.8 (31.5, 37.5)	0.0389
HDL1-apoA-II	2.4 (1.9, 3.5)	2.5 (2.1, 4.1)	2.1 (1.6, 3.0)	0.0058
HDL2-apoA-II	3.9 (3.2, 4.6)	4.0 (3.5, 4.6)	3.7 (3.0, 4.5)	0.1107

Variable (mg/dL)	All (N=130)	HV (N=65)	MS (N=65)	p
HDL3-apoA-II	7.3 (6.5, 8.2)	7.3 (6.9, 8.1)	7.1 (6.3, 8.4)	0.7392
HDL4-apoA-II	19.8 (17.5, 22.6)	20.9 (18.4, 23.2)	19.2 (17.0, 21.9)	0.0600

Data are presented as median (q1, q3). Differences between HVs and patients with MS were tested using the Mann-Whitney U test. *p*-values < 0.0005 are considered statistically significant after a Bonferroni correction for multiple testing and are depicted in bold. ApoA-I, apolipoprotein A-I; apoA-II, apolipoprotein A-II; apoB, apolipoprotein B; C, cholesterol; FC, free cholesterol; HV, healthy volunteer; HDL, high-density lipoprotein; IDL, intermediate-density lipoprotein; LDL, low-density lipoprotein; MS, metabolic syndrome patient; PL, phospholipid; TG, triglyceride; VLDL, very low-density lipoprotein.
