# Supplementary material for: Serum Levels of Adiponectin Are Strongly Associated with Lipoprotein Subclasses in Healthy Volunteers but Not in Patients with Metabolic Syndrome
Source: Int J Mol Sci. 2024 May 6;25(9):5050. doi: 10.3390/ijms25095050 (PMC11084877; doi:10.3390/ijms25095050)
Supplement: Supplementary file 1 [file ijms-25-05050-s001.zip › Table S3.pdf]

Table S3. Partial correlation analyses of serum levels of adiponectin with selected VLDL, IDL, and LDL parameters in HVs.

	Adiponectin (μg/mL)							
	Model 1		Model 2		Model 3		Model 4	
**Variable (mg/dL)**	**r**	**p**	**r**	**p**	**r**	**p**	**r**	**p**
**VLDL**								
VLDL-C	-0.38	0.0025	-0.37	0.0030	-0.36	0.0042	-0.29	0.0201
VLDL1-C	-0.35	0.0058	-0.35	0.0063	-0.33	0.0091	-0.27	0.0360
VLDL3-C	-0.42	0.0006	-0.38	0.0028	-0.41	0.0010	-0.35	0.0057
VLDL-FC	-0.37	0.0028	-0.42	0.0008	-0.36	0.0044	-0.30	0.0189
VLDL1-FC	-0.40	0.0011	-0.37	0.0032	-0.39	0.0019	-0.32	0.0101
VLDL2-FC	-0.38	0.0026	-0.40	0.0013	-0.36	0.0044	-0.29	0.0224
VLDL3-FC	-0.38	0.0021	-0.38	0.0025	-0.36	0.0036	-0.30	0.0164
VLDL-TG	-0.41	0.0010	-0.41	0.0011	-0.39	0.0015	-0.33	0.0087
VLDL1-TG	-0.36	0.0043	-0.36	0.0039	-0.35	0.0052	-0.29	0.0246
VLDL2-TG	-0.40	0.0011	-0.40	0.0013	-0.39	0.0019	-0.32	0.0102
VLDL3-TG	-0.40	0.0015	-0.39	0.0017	-0.38	0.0024	-0.32	0.0122
VLDL-PL	-0.40	0.0014	-0.40	0.0016	-0.38	0.0023	-0.32	0.0119
VLDL1-PL	-0.41	0.0009	-0.41	0.0010	-0.39	0.0015	-0.33	0.0083
VLDL2-PL	-0.42	0.0007	-0.41	0.0009	-0.40	0.0013	-0.33	0.0080
VLDL3-PL	-0.41	0.0009	-0.41	0.0011	-0.40	0.0015	-0.33	0.0084
VLDL-apoB	-0.39	0.0019	-0.39	0.0021	-0.37	0.0033	-0.30	0.0160
**IDL**								
IDL-FC	-0.41	0.0009	-0.41	0.0011	-0.40	0.0014	-0.33	0.0088
IDL-TG	-0.34	0.0074	-0.34	0.0075	-0.32	0.0106	-0.25	0.0468
**LDL**								
LDL4-C	-0.30	0.0184	-0.29	0.0218	-0.31	0.0154	-0.27	0.0308
LDL5-C	-0.41	0.0009	-0.41	0.0011	-0.41	0.0010	-0.38	0.0025
LDL5-FC	-0.35	0.0048	-0.35	0.0055	-0.35	0.0057	-0.34	0.0074
LDL5-TG	-0.37	0.0027	-0.37	0.0032	-0.35	0.0051	-0.28	0.0292
LDL4-PL	-0.33	0.0095	-0.32	0.0116	-0.33	0.0083	-0.30	0.0186
LDL5-PL	-0.43	0.0005	-0.42	0.0007	-0.42	0.0007	-0.39	0.0017
LDL4-apoB	-0.36	0.0036	-0.36	0.0046	-0.37	0.0033	-0.32	0.0106
LDL5-apoB	-0.42	0.0006	-0.42	0.0007	-0.42	0.0008	-0.38	0.0024

Spearman partial correlation analyses were used to evaluate associations of the serum levels of adiponectin with the serum levels of lipoproteins in HVs, while accounting for covariates. *p*-values < 0.0005 are considered statistically significant after a Bonferroni correction for multiple testing. Model 1: Adjusted for age, sex, and BMI. Model 2: Adjusted for age, sex, BMI, and CRP. Model 3: Adjusted for age, sex, and waist circumference. Model 4: Adjusted for age, sex, and MetSSS. ApoB, apolipoprotein B; BMI, body mass index; C, cholesterol; FC, free cholesterol; HV, healthy volunteer; IDL, intermediate-density lipoprotein; LDL, low-density lipoprotein; MetSSS, metabolic syndrome severity score; PL, phospholipid; r, Spearman's correlation coefficient; TG, triglyceride, VLDL, very low-density lipoprotein.
