# Supplementary material for: Serum Levels of Adiponectin Are Strongly Associated with Lipoprotein Subclasses in Healthy Volunteers but Not in Patients with Metabolic Syndrome
Source: Int J Mol Sci. 2024 May 6;25(9):5050. doi: 10.3390/ijms25095050 (PMC11084877; doi:10.3390/ijms25095050)
Supplement: Supplementary file 1 [file ijms-25-05050-s001.zip › Table S4.pdf]

Table S4. Partial correlation analyses of serum levels of adiponectin with serum levels of LDL and HDL in patients with MS.

Variable (mg/dL)	Adiponectin (µg/mL)							
	Model 1		Model 2		Model 3		Model 4	
	r	p	r	p	r	p	r	p
LDL1-PL	0.38	0.0025	0.38	0.0026	0.38	0.0026	0.38	0.0022
HDL-PL	0.38	0.0024	0.38	0.0027	0.38	0.0023	0.38	0.0025

Spearman partial correlation analyses were used to evaluate associations of the serum levels of adiponectin with the serum levels LDL1-PL and HDL-PL in patients with MS, while accounting for covariates. *p*-values < 0.0005 are considered statistically significant after a Bonferroni correction for multiple testing. Model 1: Adjusted for age, sex, and BMI. Model 2: Adjusted for age, sex, BMI, and CRP. Model 3: Adjusted for age, sex, and waist circumference. Model 4: Adjusted for age, sex, and MetSSS. HDL, high-density lipoprotein; LDL, MetSSS, metabolic syndrome severity score; PL, phospholipid; r, Spearman's correlation coefficient.
