# Supplementary material for: Serum Levels of Adiponectin Are Strongly Associated with Lipoprotein Subclasses in Healthy Volunteers but Not in Patients with Metabolic Syndrome
Source: Int J Mol Sci. 2024 May 6;25(9):5050. doi: 10.3390/ijms25095050 (PMC11084877; doi:10.3390/ijms25095050)
Supplement: Supplementary file 1 [file ijms-25-05050-s001.zip › Table S5.pdf]

Table S5. Differences in serum levels of lipoprotein classes and subclasses between HVs and patients with MS with low and high adiponectin levels.

	HV			MS		
**Variable (mg/dL)**	**Low adiponectin (N=33)**	**High adiponectin (N=32)**	**p-value**	**Low adiponectin (N=33)**	**High adiponectin (N=32)**	**p-value**
**VLDL**						
VLDL-C	23.21 (14.70, 26.92)	13.52 (9.53, 17.75)	0.0005	24.73 (16.59, 38.00)	23.39 (13.08, 40.59)	0.7628
VLDL1-C	7.76 (4.85, 10.13)	4.20 (2.93, 5.60)	0.0006	9.50 (5.97, 15.88)	6.95 (4.53, 14.49)	0.5417
VLDL2-C	3.12 (2.04, 4.16)	1.82 (1.27, 2.73)	0.0010	3.20 (2.09, 5.94)	3.21 (1.48, 6.52)	0.7035
VLDL3-C	4.26 (2.44, 4.85)	2.03 (1.24, 3.35)	**0.0003**	4.55 (2.82, 7.15)	4.89 (1.97, 7.93)	0.8337
VLDL4-C	5.55 (4.06, 6.86)	3.83 (2.08, 5.14)	0.0033	5.97 (4.41, 8.32)	6.50 (3.99, 8.92)	0.7930
VLDL5-C	1.15 (0.75, 1.58)	1.14 (0.95, 1.48)	0.5815	1.04 (0.68, 1.51)	1.28 (0.92, 1.77)	0.1470
VLDL-FC	9.74 (6.69, 11.96)	6.49 (4.85, 7.89)	0.0007	10.79 (8.15, 16.00)	10.70 (6.46, 15.77)	0.7779
VLDL1-FC	2.70 (1.80, 3.83)	1.14 (0.62, 1.75)	**< 0.0001**	3.24 (2.32, 5.48)	2.83 (1.08, 4.53)	0.3348
VLDL2-FC	1.18 (0.85, 1.84)	0.51 (0.32, 0.98)	0.0006	1.54 (0.84, 2.57)	1.38 (0.42, 2.69)	0.5461
VLDL3-FC	1.74 (0.97, 2.37)	0.80 (0.53, 1.21)	0.0009	2.10 (1.39, 3.34)	2.13 (0.77, 3.39)	0.6134
VLDL4-FC	2.40 (1.73, 3.10)	1.71 (0.98, 2.24)	0.0031	2.71 (2.11, 3.72)	3.24 (1.75, 4.45)	0.6042
VLDL5-FC	0.53 (0.35, 0.89)	0.62 (0.41, 0.88)	0.7828	0.65 (0.44, 1.02)	0.81 (0.55, 0.99)	0.5461
VLDL-TG	73.67 (52.61, 97.97)	40.86 (31.49, 58.49)	**< 0.0001**	92.33 (68.83, 139.95)	92.58 (47.12, 123.98)	0.3186
VLDL1-TG	30.04 (22.83, 48.25)	14.92 (9.28, 25.83)	**< 0.0001**	48.34 (33.54, 77.59)	43.56 (20.97, 61.16)	0.2325
VLDL2-TG	10.59 (7.34, 15.92)	5.94 (4.10, 9.05)	**0.0003**	13.29 (9.34, 20.65)	13.16 (6.85, 22.29)	0.4311
VLDL3-TG	9.67 (6.52, 14.08)	5.22 (3.92, 8.39)	0.0007	12.00 (8.46, 18.45)	13.02 (5.32, 19.86)	0.5997
VLDL4-TG	8.66 (6.47, 10.19)	5.77 (4.32, 7.87)	0.0029	10.31 (8.80, 13.64)	10.50 (6.49, 13.55)	0.7379
VLDL5-TG	2.44 (2.07, 3.01)	2.60 (2.24, 2.84)	0.6366	2.92 (2.48, 3.75)	3.06 (2.70, 3.48)	0.5075
VLDL-PL	20.36 (13.84, 25.65)	12.55 (9.71, 16.34)	**0.0003**	22.76 (18.22, 34.68)	22.77 (12.32, 31.99)	0.3722
VLDL1-PL	5.67 (4.06, 7.96)	2.41 (1.26, 3.94)	**< 0.0001**	7.82 (5.07, 12.30)	6.85 (3.35, 10.27)	0.2325
VLDL2-PL	2.89 (2.18, 4.37)	1.54 (1.12, 2.38)	**0.0002**	3.49 (2.40, 5.32)	3.25 (1.67, 5.71)	0.3549
VLDL3-PL	3.64 (2.31, 5.00)	1.83 (1.38, 2.82)	**0.0003**	4.38 (3.16, 6.47)	4.65 (1.96, 7.04)	0.4909
VLDL4-PL	4.74 (3.44, 5.24)	3.27 (1.99, 4.15)	0.0015	5.17 (4.27, 6.87)	5.71 (3.43, 7.19)	0.9216
VLDL5-PL	1.46 (1.15, 2.04)	1.42 (1.13, 1.74)	0.5996	1.67 (1.09, 2.12)	1.83 (1.49, 2.16)	0.3617
VLDL-apoB	8.02 (5.69, 10.18)	5.15 (3.40, 6.61)	0.0007	9.20 (7.46, 13.36)	10.00 (5.54, 13.35)	0.7280
**IDL**						
IDL-C	17.97 (12.73, 20.55)	11.59 (8.80, 15.63)	0.0007	17.50 (14.04, 25.22)	21.90 (11.66, 27.79)	0.5906
IDL-FC	5.04 (3.49, 5.95)	3.14 (2.40, 4.48)	0.0005	4.90 (3.80, 7.04)	6.01 (3.23, 8.00)	0.6698

	HV			MS		
Variable (mg/dL)	Low adiponectin (N=33)	High adiponectin (N=32)	p-value	Low adiponectin (N=33)	High adiponectin (N=32)	p-value
IDL-TG	9.55 (6.45, 15.53)	5.95 (3.63, 7.68)	0.0005	13.83 (9.85, 23.45)	12.84 (6.04, 21.74)	0.5331
IDL-PL	8.42 (6.75, 10.68)	6.45 (5.54, 7.79)	0.0007	9.42 (6.47, 11.74)	9.96 (6.50, 12.80)	0.9164
IDL-apoB	6.54 (4.50, 7.23)	4.42 (3.21, 5.57)	0.0013	6.59 (4.99, 8.57)	7.56 (5.08, 9.26)	0.6650
**LDL**						
LDL-C	145.49 (123.08, 162.27)	132.32 (121.78, 152.50)	0.2704	111.23 (79.10, 134.74)	119.64 (103.46, 152.17)	0.1280
LDL1-C	29.09 (25.01, 36.00)	32.46 (27.43, 37.55)	0.1643	22.07 (16.38, 26.45)	29.09 (24.14, 36.66)	**0.0004**
LDL2-C	22.62 (15.92, 29.65)	26.53 (25.29, 32.60)	0.0152	15.42 (10.13, 20.19)	19.32 (14.17, 22.47)	0.0856
LDL3-C	22.44 (19.82, 29.08)	24.69 (21.73, 29.09)	0.3937	15.27 (7.12, 20.38)	19.91 (13.44, 24.59)	0.0346
LDL4-C	22.49 (17.58, 27.92)	17.82 (14.35, 21.82)	0.0030	17.07 (8.94, 21.23)	18.35 (15.32, 24.86)	0.1894
LDL5-C	21.48 (15.29, 26.40)	12.82 (10.32, 16.42)	**< 0.0001**	17.25 (12.77, 23.77)	17.56 (13.10, 22.68)	0.8440
LDL6-C	20.21 (18.11, 28.63)	17.12 (15.25, 20.89)	0.0022	22.38 (17.55, 30.23)	21.36 (16.02, 27.30)	0.3583
LDL-FC	43.98 (37.84, 48.24)	41.37 (38.37, 47.17)	0.8491	35.25 (26.40, 40.63)	38.05 (33.11, 46.61)	0.0615
LDL1-FC	9.25 (8.12, 10.93)	9.73 (8.72, 11.48)	0.2299	7.51 (6.10, 8.46)	9.54 (7.79, 11.70)	**0.0003**
LDL2-FC	7.81 (5.32, 9.39)	8.78 (8.40, 10.63)	0.0092	5.56 (4.64, 7.30)	6.77 (5.61, 8.04)	0.0369
LDL3-FC	7.63 (6.10, 9.09)	7.95 (7.54, 9.54)	0.0930	5.74 (3.80, 6.86)	6.85 (5.24, 7.84)	0.0192
LDL4-FC	6.79 (6.07, 7.98)	5.89 (4.94, 7.01)	0.0341	5.48 (3.72, 6.67)	6.13 (5.05, 7.63)	0.0702
LDL5-FC	6.16 (5.11, 7.18)	4.46 (3.75, 5.53)	**0.0002**	5.38 (4.43, 6.67)	5.30 (4.77, 6.99)	0.6987
LDL6-FC	6.16 (5.32, 7.08)	5.47 (4.81, 6.18)	0.0163	6.30 (5.59, 7.67)	6.49 (4.99, 7.33)	0.4992
LDL-TG	20.79 (18.07, 24.52)	18.34 (16.05, 22.08)	0.0983	22.36 (20.16, 26.93)	24.54 (21.71, 30.71)	0.1985
LDL1-TG	6.38 (5.28, 7.49)	6.29 (4.74, 7.59)	0.7628	7.03 (5.84, 8.16)	7.97 (6.71, 9.66)	0.0224
LDL2-TG	2.64 (2.21, 2.93)	2.79 (2.15, 3.34)	0.2848	2.36 (2.00, 2.71)	2.90 (2.36, 3.30)	0.0205
LDL3-TG	2.51 (1.92, 2.82)	2.60 (2.22, 3.16)	0.1622	2.23 (1.75, 2.68)	2.73 (2.01, 3.14)	0.0754
LDL4-TG	2.80 (1.91, 3.20)	1.90 (1.48, 2.69)	0.0080	2.85 (1.99, 3.48)	2.98 (2.65, 4.25)	0.2619
LDL5-TG	2.80 (1.94, 3.61)	1.64 (1.12, 2.13)	**0.0003**	3.11 (2.35, 4.12)	3.00 (2.16, 4.31)	0.9216
LDL6-TG	2.81 (2.51, 3.86)	2.68 (2.20, 3.10)	0.1471	3.98 (2.80, 4.86)	3.87 (2.89, 4.41)	0.3154
LDL-PL	79.41 (67.88, 87.13)	73.41 (67.79, 83.31)	0.4159	62.46 (47.06, 74.91)	67.97 (60.71, 83.94)	0.0722
LDL1-PL	16.25 (13.73, 19.15)	17.80 (14.91, 20.83)	0.1545	12.73 (10.76, 14.56)	16.39 (14.03, 20.30)	**0.0003**
LDL2-PL	12.52 (8.79, 15.62)	14.56 (13.58, 17.45)	0.0079	8.62 (6.16, 11.04)	10.79 (7.78, 12.59)	0.0483
LDL3-PL	12.12 (10.79, 15.59)	13.42 (11.83, 15.81)	0.2819	8.77 (4.68, 11.26)	11.11 (8.16, 13.57)	0.0253
LDL4-PL	12.03 (9.73, 14.61)	9.69 (7.90, 11.90)	0.0018	9.56 (5.42, 11.50)	10.36 (8.86, 13.77)	0.1683
LDL5-PL	11.74 (8.41, 13.79)	7.33 (5.85, 9.11)	**< 0.0001**	9.59 (7.52, 12.60)	9.69 (7.59, 12.63)	0.9112

	HV			MS		
**Variable (mg/dL)**	**Low adiponectin (N=33)**	**High adiponectin (N=32)**	**p-value**	**Low adiponectin (N=33)**	**High adiponectin (N=32)**	**p-value**
LDL6-PL	11.82 (10.50, 14.81)	10.38 (9.39, 12.01)	0.0120	12.65 (10.70, 16.26)	12.64 (9.95, 14.76)	0.3865
LDL-apoB	86.78 (74.95, 100.04)	77.95 (71.55, 91.22)	0.0682	72.84 (58.44, 91.11)	82.29 (73.03, 94.55)	0.1895
LDL1-apoB	15.10 (13.04, 17.81)	16.62 (13.74, 19.62)	0.1399	12.21 (11.12, 14.41)	15.71 (13.35, 19.38)	**0.0003**
LDL2-apoB	12.28 (9.20, 15.54)	14.59 (13.34, 16.94)	0.0160	9.64 (6.80, 11.51)	10.91 (8.62, 13.28)	0.0652
LDL3-apoB	13.47 (11.65, 16.42)	14.27 (12.33, 16.43)	0.4505	10.00 (5.49, 12.43)	11.77 (9.26, 15.02)	0.0224
LDL4-apoB	14.42 (11.66, 17.17)	10.72 (8.68, 13.62)	0.0005	12.30 (7.40, 15.15)	12.37 (10.28, 16.86)	0.1642
LDL5-apoB	14.98 (10.46, 18.04)	8.94 (6.94, 11.52)	**< 0.0001**	12.75 (9.92, 18.17)	13.00 (9.92, 17.21)	0.7379
LDL6-apoB	16.84 (15.10, 22.74)	13.82 (11.83, 16.47)	0.0011	18.97 (15.56, 25.90)	17.19 (13.36, 23.33)	0.4010
**HDL**						
HDL-C	59.00 (53.04, 65.65)	71.82 (64.69, 82.81)	**< 0.0001**	52.23 (45.11, 58.58)	54.36 (48.97, 66.09)	0.1643
HDL1-C	16.64 (13.67, 18.38)	25.88 (18.79, 32.20)	**< 0.0001**	14.52 (12.09, 17.96)	16.65 (13.96, 21.02)	0.0652
HDL2-C	8.47 (7.71, 8.93)	10.91 (9.57, 13.31)	**< 0.0001**	7.69 (6.50, 8.80)	8.61 (7.47, 10.07)	0.0394
HDL3-C	11.26 (10.46, 13.13)	13.13 (11.61, 14.06)	0.0051	10.36 (9.25, 11.41)	10.75 (9.72, 12.50)	0.1471
HDL4-C	23.02 (18.24, 25.09)	21.59 (18.05, 23.98)	0.2704	18.42 (15.99, 22.09)	19.16 (16.01, 22.71)	0.8956
HDL-FC	14.26 (12.86, 15.62)	18.34 (16.23, 21.00)	**< 0.0001**	12.79 (11.43, 14.61)	13.87 (12.84, 16.60)	0.0111
HDL1-FC	5.01 (4.36, 5.45)	7.06 (5.43, 8.45)	**< 0.0001**	4.18 (3.37, 5.07)	4.63 (4.14, 5.84)	0.0206
HDL2-FC	2.47 (1.97, 2.85)	2.85 (2.62, 3.41)	0.0006	2.17 (1.92, 2.69)	2.38 (2.17, 2.73)	0.0652
HDL3-FC	2.91 (2.39, 3.24)	3.07 (2.71, 3.47)	0.1526	2.31 (1.93, 2.97)	2.66 (2.27, 3.17)	0.0787
HDL4-FC	5.02 (4.21, 5.66)	4.73 (3.76, 5.66)	0.3828	4.04 (3.63, 4.83)	4.67 (3.89, 5.23)	0.1765
HDL-TG	9.66 (8.66, 11.02)	10.14 (8.66, 12.57)	0.3381	10.72 (8.60, 12.98)	12.79 (10.34, 14.06)	0.0330
HDL1-TG	2.71 (2.09, 3.47)	3.84 (2.70, 5.06)	0.0041	2.81 (2.20, 3.81)	3.75 (3.44, 5.44)	0.0065
HDL2-TG	1.56 (1.27, 2.02)	1.67 (1.53, 2.20)	0.2968	1.85 (1.51, 2.42)	2.23 (1.83, 2.69)	0.0341
HDL3-TG	2.14 (1.77, 2.73)	2.16 (1.56, 2.41)	0.3756	2.60 (1.95, 3.07)	2.71 (2.15, 3.25)	0.2878
HDL4-TG	3.60 (3.34, 4.12)	2.83 (2.10, 3.73)	0.0012	3.74 (3.22, 4.62)	3.82 (3.38, 4.74)	0.5861
HDL-PL	80.86 (74.34, 88.47)	96.57 (89.80, 109.66)	**< 0.0001**	72.70 (63.47, 80.93)	80.45 (69.46, 89.27)	0.0537
HDL1-PL	19.04 (16.74, 22.11)	31.79 (22.25, 39.44)	**< 0.0001**	18.38 (14.18, 20.57)	20.77 (17.18, 26.47)	0.0217
HDL2-PL	12.98 (11.66, 14.46)	16.55 (14.36, 20.15)	**< 0.0001**	12.01 (10.23, 13.84)	14.17 (11.29, 16.84)	0.0294
HDL3-PL	17.99 (16.12, 20.19)	20.30 (18.27, 21.72)	0.0050	16.57 (15.23, 17.92)	18.38 (15.41, 19.81)	0.1489
HDL4-PL	30.04 (26.35, 33.51)	29.46 (26.19, 32.64)	0.4584	26.64 (22.58, 30.71)	26.23 (24.08, 30.64)	0.6508
HDL-apoA-I	159.24 (147.25, 171.76)	181.45 (167.49, 192.62)	**0.0003**	148.31 (134.79, 164.49)	152.44 (142.31, 173.08)	0.0643
HDL1-apoA-I	23.55 (19.48, 27.90)	41.00 (27.07, 53.20)	**< 0.0001**	22.15 (17.59, 27.38)	26.67 (20.94, 33.67)	0.0284

	HV			MS		
Variable (mg/dL)	Low adiponectin (N=33)	High adiponectin (N=32)	p-value	Low adiponectin (N=33)	High adiponectin (N=32)	p-value
HDL2-apoA-I	18.04 (16.56, 19.81)	22.64 (19.47, 25.75)	**0.0001**	17.24 (15.14, 18.83)	18.38 (15.93, 21.73)	0.1939
HDL3-apoA-I	28.76 (27.23, 33.09)	32.58 (29.62, 35.43)	0.0113	27.22 (24.66, 31.10)	30.27 (26.00, 33.36)	0.1123
HDL4-apoA-I	86.74 (73.91, 92.49)	79.49 (69.91, 88.35)	0.1010	73.68 (68.33, 83.94)	77.01 (70.09, 87.02)	0.4388
HDL-apoA-II	36.22 (32.78, 39.60)	35.31 (33.68, 38.33)	0.4466	33.77 (30.77, 36.85)	34.06 (31.61, 38.53)	0.6180
HDL1-apoA-II	2.36 (1.88, 2.81)	3.85 (2.35, 4.95)	0.0008	2.01 (1.53, 2.41)	2.27 (1.82, 3.28)	0.2054
HDL2-apoA-II	3.91 (3.49, 4.20)	4.34 (3.62, 4.84)	0.0261	3.51 (2.76, 4.20)	3.80 (3.01, 4.72)	0.5593
HDL3-apoA-II	7.48 (6.96, 8.20)	7.21 (6.84, 7.86)	0.2008	7.08 (6.26, 8.19)	7.38 (6.44, 8.73)	0.5288
HDL4-apoA-II	22.38 (18.96, 24.56)	19.00 (16.97, 22.14)	0.0057	19.23 (17.35, 22.50)	19.08 (16.95, 21.72)	0.6746

Data are presented as median (q1, q3). Differences between HVs and MS patients with low and high adiponectin were tested using the Mann-Whitney U test. Adiponectin levels in HV and MS below the median (15.1 μg/mL and 13.0 μg/mL, respectively) were defined as low and those ≥15.1 μg/mL and ≥13.0 μg/mL, respectively, as high adiponectin. *p*-values < 0.0005 are considered statistically significant after a Bonferroni correction for multiple testing and are depicted in bold. ApoA-I, apolipoprotein A-I; apoA-II, apolipoprotein A-II; apoB, apolipoprotein B; BMI, body mass index; C, cholesterol; FC, free cholesterol; HV, healthy volunteer; HDL, high-density lipoprotein; IDL, intermediate-density lipoprotein; LDL, low-density lipoprotein; MS, metabolic syndrome patient; PL, phospholipid; r, Spearman's correlation coefficient; TG, triglyceride; VLDL, very low-density lipoprotein.
