# Supplementary material for: Serum Levels of Adiponectin Are Strongly Associated with Lipoprotein Subclasses in Healthy Volunteers but Not in Patients with Metabolic Syndrome
Source: Int J Mol Sci. 2024 May 6;25(9):5050. doi: 10.3390/ijms25095050 (PMC11084877; doi:10.3390/ijms25095050)
Supplement: Supplementary file 1 [file ijms-25-05050-s001.zip › Table S6.pdf]

Table S6. OPLS-DA VIP scores in HVs and patients with MS.

HV		MS	
Variable	VIP score	Variable	VIP score
LDL5-apoB	1,49338724	LDL1-PL	2,35982012
HDL1-PL	1,47992575	LDL1-FC	2,30379512
LDL5-PL	1,47566421	LDL1-C	2,29319643
LDL5-C	1,46705101	LDL1-apoB	2,26299667
HDL1-apoA-I	1,46469696	HDL-FC	1,95893553
HDL2-PL	1,44236795	HDL-apoA-I	1,78121973
HDL2-C	1,44114218	HDL-PL	1,69304879
HDL1-C	1,44044305	HDL1-PL	1,57030139
HDL-PL	1,4256839	LDL3-FC	1,51785087
HDL1-FC	1,39755129	HDL1-FC	1,48221796
HDL1-apoA-II	1,37874257	HDL-C	1,47311727
LDL5-FC	1,35480922	HDL3-C	1,43547135
HDL-FC	1,33675104	HDL1-apoA-I	1,41143951
HDL-C	1,3310263	LDL3-PL	1,37960908
HDL2-apoA-I	1,33031037	HDL3-FC	1,35390553
LDL5-TG	1,32294126	HDL3-apoA-I	1,34850803
VLDL1-PL	1,30356611	LDL3-apoB	1,34720563
HDL2-FC	1,28669509	HDL2-C	1,33606546
VLDL1-TG	1,27730687	HDL2-PL	1,33513416
VLDL1-FC	1,25048359	LDL3-C	1,33263027
VLDL-TG	1,25030428	HDL2-FC	1,31444091
LDL6-apoB	1,2295365	HDL3-PL	1,2853978
HDL4-TG	1,20751233	HDL1-C	1,25099635
VLDL2-PL	1,20618747	LDL4-FC	1,21203434
LDL4-apoB	1,19910672	LDL6-TG	1,21172135
HDL-apoA-I	1,18720691	LDL6-FC	1,19834965
VLDL-PL	1,18435399	LDL-FC	1,18187724
VLDL-apoB	1,16834825	VLDL5-C	1,17045164
LDL6-C	1,16258369	LDL2-PL	1,1104765
VLDL2-TG	1,14795806	HDL2-apoA-I	1,09141746
VLDL1-C	1,14712528	LDL6-PL	1,08817203
IDL-TG	1,14113406	LDL4-PL	1,07387001
VLDL-FC	1,13525621	LDL6-C	1,05741998
VLDL-C	1,13005069	LDL-PL	1,0201385
VLDL3-PL	1,11876458	LDL2-FC	1,00532741
IDL-FC	1,1110435	LDL4-C	1,00524318
VLDL2-FC	1,10760696	LDL4-apoB	0,99527422
LDL4-PL	1,10697273	LDL6-apoB	0,96850053
LDL4-C	1,10068124	LDL2-C	0,94173643
VLDL3-C	1,09755611	LDL2-apoB	0,93081073
IDL-PL	1,09470529	VLDL5-PL	0,92650281
VLDL2-C	1,09335876	LDL-C	0,90164187
VLDL3-FC	1,08042699	HDL4-FC	0,88854581
IDL-C	1,06596238	LDL3-TG	0,85713478
VLDL3-TG	1,0571365	HDL4-apoA-I	0,84730064
HDL4-apoA-II	1,05165916	LDL2-TG	0,79064889
LDL6-PL	1,04234319	VLDL1-TG	0,68913437
HDL1-TG	1,02428417	VLDL1-PL	0,67928889
IDL-apoB	1,02311745	HDL4-PL	0,66300433
LDL2-FC	1,01667751	HDL-apoA-II	0,65519579
LDL2-PL	1,00796734	HDL1-apoA-II	0,64531406
LDL4-TG	0,99808297	VLDL-TG	0,63706954
LDL2-C	0,98914996	VLDL2-TG	0,61849854
LDL6-FC	0,97781325	IDL-TG	0,60782994

VLDL4-PL	0,9716503	VLDL2-PL	0,58701437
HDL2-apoA-II	0,95653986	LDL5-TG	0,53563375
VLDL4-FC	0,93242494	LDL-apoB	0,51593338
VLDL4-TG	0,92659675	VLDL1-FC	0,51102135
LDL2-apoB	0,92443915	VLDL3-TG	0,50867148
VLDL4-C	0,918839	VLDL5-FC	0,50287656
LDL6-TG	0,87322415	LDL4-TG	0,49927385
LDL4-FC	0,85180907	HDL3-apoA-II	0,48955205
HDL3-C	0,81960819	VLDL5-TG	0,48110384
HDL4-apoA-I	0,79239015	VLDL3-PL	0,47883026
HDL3-PL	0,77460414	LDL5-apoB	0,45370801
LDL-apoB	0,73203295	VLDL-PL	0,45166542
HDL3-apoA-I	0,7176559	VLDL4-TG	0,44605848
LDL-TG	0,71164004	HDL-TG	0,43621322
HDL4-PL	0,61271026	HDL2-apoA-II	0,38147476
LDL1-PL	0,58731352	VLDL1-C	0,37692477
LDL1-apoB	0,56646299	HDL1-TG	0,36808604
LDL3-FC	0,56466196	HDL4-C	0,36734389
LDL1-FC	0,56427465	VLDL3-FC	0,35730159
HDL4-C	0,56261628	HDL2-TG	0,34669962
HDL4-FC	0,55935609	LDL5-PL	0,34181715
LDL1-C	0,55838663	LDL5-C	0,33589411
HDL3-TG	0,46134669	HDL4-TG	0,32766006
LDL2-TG	0,43524529	VLDL-apoB	0,3241261
HDL2-TG	0,42825967	VLDL2-FC	0,3059116
LDL-C	0,40856549	VLDL4-FC	0,29235047
HDL3-FC	0,3983612	VLDL4-C	0,26962256
LDL3-TG	0,3686273	HDL4-apoA-II	0,26251542
LDL-PL	0,34317031	IDL-PL	0,25391775
HDL-apoA-II	0,33137242	VLDL2-C	0,23557715
LDL3-PL	0,3309615	VLDL-FC	0,2247766
LDL3-C	0,30205504	HDL3-TG	0,1987866
VLDL5-PL	0,29176307	VLDL-C	0,19499652
HDL-TG	0,24661595	VLDL3-C	0,18023826
LDL3-apoB	0,23962632	LDL-TG	0,1338694
HDL3-apoA-II	0,20904614	LDL1-TG	0,13368183
LDL1-TG	0,16266827	LDL5-FC	0,12519715
LDL-FC	0,14409132	VLDL4-PL	0,10608908
VLDL5-C	0,05658013	IDL-C	0,05822336
VLDL5-FC	0,0548439	IDL-apoB	0,05590506
VLDL5-TG	0,02097973	IDL-FC	0,00777274
