# Supplementary material for: Serum Levels of Adiponectin Are Strongly Associated with Lipoprotein Subclasses in Healthy Volunteers but Not in Patients with Metabolic Syndrome
Source: Int J Mol Sci. 2024 May 6;25(9):5050. doi: 10.3390/ijms25095050 (PMC11084877; doi:10.3390/ijms25095050)
Supplement: Supplementary file 1 [file ijms-25-05050-s001.zip › Table S7.pdf]

Table S7. Logistic regression analyses to assess associations of lipoproteins and their subclasses with high vs. low adiponectin, separately in HVs and patients with MS.

	Adiponectin ≥15.1 μg/mL		Adiponectin ≥13.0 μg/mL	
	HV		MS	
**Variable (mg/dL)**	**OR (95% CI)**	**p-value**	**OR (95% CI)**	**p-value**
**VLDL**				
VLDL-C	0.90 (0.84-0.96)	0.0026	1.00 (0.98-1.02)	0.7520
VLDL1-C	0.75 (0.60-0.88)	0.0023	0.99 (0.95-1.03)	0.6661
VLDL2-C	0.56 (0.36-0.80)	0.0047	0.98 (0.86-1.10)	0.7038
VLDL3-C	0.64 (0.45-0.84)	0.0039	0.98 (0.87-1.10)	0.7573
VLDL4-C	0.72 (0.55-0.92)	0.0133	1.01 (0.87-1.17)	0.8715
VLDL5-C	1.24 (0.49-3.27)	0.6479	1.62 (0.73-3.79)	0.2474
VLDL-FC	0.77 (0.63-0.90)	0.0032	0.99 (0.93-1.04)	0.6865
VLDL1-FC	0.40 (0.22-0.64)	0.0007	0.95 (0.83-1.08)	0.4625
VLDL2-FC	0.30 (0.12-0.62)	0.0041	0.95 (0.75-1.18)	0.6460
VLDL3-FC	0.38 (0.18-0.69)	0.0045	0.94 (0.75-1.16)	0.5792
VLDL4-FC	0.49 (0.28-0.81)	0.0085	1.03 (0.80-1.35)	0.8022
VLDL5-FC	1.15 (0.26-5.11)	0.8469	1.33 (0.58-3.67)	0.5101
VLDL-TG	0.96 (0.94-0.98)	0.0012	1.00 (0.99-1.00)	0.3564
VLDL1-TG	0.93 (0.88-0.96)	0.0008	1.00 (0.99-1.00)	0.3839
VLDL2-TG	0.84 (0.73-0.93)	0.0036	0.98 (0.94-1.02)	0.3113
VLDL3-TG	0.85 (0.74-0.94)	0.0067	0.98 (0.93-1.02)	0.3675
VLDL4-TG	0.80 (0.65-0.94)	0.0148	0.96 (0.87-1.03)	0.3014
VLDL5-TG	1.21 (0.54-2.76)	0.6413	1.08 (0.61-1.94)	0.7957
VLDL-PL	0.87 (0.79-0.94)	0.0016	0.99 (0.95-1.02)	0.4078
VLDL1-PL	0.63 (0.47-0.80)	0.0007	0.98 (0.91-1.03)	0.3840
VLDL2-PL	0.47 (0.28-0.72)	0.0020	0.92 (0.77-1.08)	0.3328
VLDL3-PL	0.57 (0.38-0.79)	0.0024	0.94 (0.80-1.07)	0.3663
VLDL4-PL	0.62 (0.42-0.86)	0.0082	0.95 (0.79-1.14)	0.6094
VLDL5-PL	0.84 (0.36-1.91)	0.6797	1.32 (0.63-2.83)	0.4638
VLDL-apoB	0.74 (0.59-0.88)	0.0025	0.98 (0.90-1.05)	0.5592
**IDL**				
IDL-C	0.85 (0.75-0.93)	0.0020	1.00 (0.96-1.04)	0.9846
IDL-FC	0.55 (0.37-0.78)	0.0015	1.00 (0.88-1.15)	0.9534
IDL-TG	0.83 (0.72-0.92)	0.0024	0.99 (0.96-1.01)	0.3900
IDL-PL	0.67 (0.51-0.85)	0.0021	0.99 (0.91-1.06)	0.6777
IDL-apoB	0.62 (0.44-0.83)	0.0028	1.00 (0.87-1.15)	0.9720
**LDL**				
LDL-C	0.99 (0.97-1.01)	0.3321	1.01 (1.00-1.03)	0.1442
LDL1-C	1.06 (0.99-1.13)	0.0941	1.13 (1.06-1.24)	0.0016
LDL2-C	1.12 (1.04-1.21)	0.0051	1.07 (0.99-1.16)	0.0809
LDL3-C	1.04 (0.96-1.13)	0.3406	1.07 (1.01-1.14)	0.0403
LDL4-C	0.87 (0.78-0.95)	0.0033	1.04 (0.99-1.10)	0.1320
LDL5-C	0.81 (0.71-0.90)	0.0003	0.98 (0.92-1.05)	0.6169
LDL6-C	0.86 (0.76-0.95)	0.0059	0.97 (0.92-1.01)	0.1882
LDL-FC	0.99 (0.93-1.06)	0.8593	1.05 (1.00-1.11)	0.0624
LDL1-FC	1.21 (0.98-1.55)	0.0917	1.56 (1.21-2.12)	0.0016
LDL2-FC	1.47 (1.16-1.96)	0.0037	1.31 (1.00-1.76)	0.0596
LDL3-FC	1.33 (1.00-1.83)	0.0630	1.35 (1.06-1.77)	0.0215
LDL4-FC	0.67 (0.46-0.94)	0.0289	1.23 (0.99-1.56)	0.0709
LDL5-FC	0.49 (0.31-0.72)	0.0008	0.99 (0.77-1.27)	0.9096
LDL6-FC	0.61 (0.38-0.90)	0.0226	0.84 (0.65-1.05)	0.1537

LDL-TG	0.91 (0.81-1.01)	0.0969	1.00 (0.96-1.05)	0.9031
LDL1-TG	1.00 (0.77-1.29)	0.9724	1.02 (0.92-1.15)	0.7148
LDL2-TG	1.53 (0.81-3.05)	0.1980	1.40 (0.88-2.45)	0.1822
LDL3-TG	1.79 (0.84-4.02)	0.1422	1.71 (0.93-3.42)	0.1016
LDL4-TG	0.44 (0.23-0.78)	0.0091	1.17 (0.84-1.69)	0.3609
LDL5-TG	0.32 (0.16-0.57)	0.0005	0.91 (0.67-1.20)	0.5208
LDL6-TG	0.61 (0.33-1.05)	0.0869	0.84 (0.62-1.05)	0.2007
LDL-PL	0.99 (0.95-1.03)	0.4981	1.02 (1.00-1.05)	0.1011
LDL1-PL	1.12 (1.00-1.29)	0.0719	1.31 (1.13-1.58)	0.0012
LDL2-PL	1.25 (1.09-1.49)	0.0039	1.17 (1.01-1.38)	0.0452
LDL3-PL	1.10 (0.93-1.30)	0.2689	1.14 (1.02-1.31)	0.0325
LDL4-PL	0.76 (0.62-0.90)	0.0033	1.09 (0.99-1.22)	0.1013
LDL5-PL	0.66 (0.51-0.80)	0.0003	0.97 (0.86-1.10)	0.6220
LDL6-PL	0.77 (0.61-0.94)	0.0161	0.93 (0.84-1.03)	0.1833
LDL-apoB	0.97 (0.94-1.00)	0.0878	1.01 (0.99-1.04)	0.2911
LDL1-apoB	1.13 (1.00-1.30)	0.0727	1.32 (1.13-1.59)	0.0012
LDL2-apoB	1.23 (1.07-1.46)	0.0068	1.15 (0.99-1.35)	0.0677
LDL3-apoB	1.06 (0.92-1.24)	0.4092	1.14 (1.02-1.30)	0.0305
LDL4-apoB	0.78 (0.65-0.90)	0.0014	1.07 (0.98-1.17)	0.1434
LDL5-apoB	0.74 (0.61-0.85)	0.0002	0.97 (0.88-1.07)	0.5590
LDL6-apoB	0.82 (0.70-0.92)	0.0041	0.97 (0.92-1.02)	0.2517
**HDL**				
HDL-C	1.11 (1.06-1.19)	0.0003	1.05 (1.00-1.10)	0.0611
HDL1-C	1.19 (1.10-1.33)	0.0003	1.10 (1.00-1.23)	0.0527
HDL2-C	1.81 (1.37-2.58)	0.0002	1.27 (1.02-1.66)	0.0478
HDL3-C	1.58 (1.16-2.23)	0.0061	1.22 (0.99-1.57)	0.0787
HDL4-C	0.93 (0.82-1.05)	0.2302	1.00 (0.92-1.09)	0.9688
HDL-FC	1.50 (1.23-1.93)	0.0003	1.32 (1.10-1.65)	0.0067
HDL1-FC	1.97 (1.40-3.04)	0.0005	1.65 (1.11-2.65)	0.0214
HDL2-FC	7.44 (2.56-27.42)	0.0008	2.36 (1.04-6.07)	0.0525
HDL3-FC	2.19 (0.82-6.24)	0.1270	1.77 (0.94-3.60)	0.0900
HDL4-FC	0.78 (0.46-1.27)	0.3198	1.19 (0.84-1.76)	0.3410
HDL-TG	1.06 (0.89-1.27)	0.5206	1.04 (0.95-1.14)	0.4144
HDL1-TG	1.66 (1.17-2.58)	0.0110	1.07 (0.91-1.32)	0.4314
HDL2-TG	1.64 (0.70-4.02)	0.2590	1.19 (0.79-1.91)	0.4240
HDL3-TG	0.57 (0.24-1.30)	0.1910	1.09 (0.73-1.67)	0.6700
HDL4-TG	0.35 (0.17-0.65)	0.0018	1.06 (0.65-1.73)	0.8173
HDL-PL	1.11 (1.06-1.17)	0.0002	1.04 (1.01-1.08)	0.0278
HDL1-PL	1.16 (1.08-1.27)	0.0002	1.12 (1.03-1.23)	0.0172
HDL2-PL	1.52 (1.25-1.96)	0.0002	1.16 (1.01-1.37)	0.0419
HDL3-PL	1.33 (1.09-1.67)	0.0083	1.11 (0.98-1.28)	0.1192
HDL4-PL	0.93 (0.83-1.04)	0.2435	1.01 (0.95-1.09)	0.6746
HDL-apoA-I	1.05 (1.03-1.09)	0.0008	1.03 (1.00-1.05)	0.0300
HDL1-apoA-I	1.10 (1.05-1.17)	0.0003	1.07 (1.01-1.14)	0.0314
HDL2-apoA-I	1.36 (1.17-1.65)	0.0003	1.10 (0.98-1.25)	0.1123
HDL3-apoA-I	1.20 (1.05-1.39)	0.0107	1.08 (1.00-1.18)	0.0775
HDL4-apoA-I	0.96 (0.91-1.00)	0.0656	1.01 (0.98-1.04)	0.4758
HDL-apoA-II	0.94 (0.82-1.07)	0.3741	1.03 (0.94-1.15)	0.5005
HDL1-apoA-II	2.40 (1.52-4.26)	0.0007	1.24 (0.86-1.86)	0.2642
HDL2-apoA-II	2.22 (1.20-4.52)	0.0172	1.10 (0.78-1.58)	0.6009
HDL3-apoA-II	0.85 (0.52-1.37)	0.5099	1.07 (0.83-1.40)	0.5931
HDL4-apoA-II	0.81 (0.68-0.93)	0.0067	0.99 (0.88-1.11)	0.8369

Results are presented as odds ratios (ORs) and 95% confidence interval (CI) per increase in one unit (mg/dL) of (A) LDL and (B) HDL parameters. HVs with adiponectin <15.1 μg/mL belong to low (N=33) and those with adiponectin ≥15.1 μg/mL to high adiponectin group (N=32). Patients with MS with adiponectin <13.0 μg/mL belong to low

(N=33) and those with adiponectin ≥13.0 μg/mL to high adiponectin group (N=32). p-values < 0.0005 are considered statistically significant after a Bonferroni correction for multiple testing. Significant parameters as well as their respective ORs and 95% CIs are depicted in red. In MS, the parameters, ORs, and 95% CIs depicted in purple are those with the highest VIP scores in OPLS-DA analysis. ApoA-I, apolipoprotein A-I; apoA-II, apolipoprotein A-II; apoB, apolipoprotein B; C, cholesterol; FC, free cholesterol; HDL, high-density lipoprotein; HV, healthy volunteer; IDL, intermediate-density lipoprotein LDL, low-density lipoprotein; MS, metabolic syndrome patient; PL, phospholipid; TG, triglyceride; VIP, variable of importance projection.
