# Supplementary material for: Serum Levels of Adiponectin Are Strongly Associated with Lipoprotein Subclasses in Healthy Volunteers but Not in Patients with Metabolic Syndrome
Source: Int J Mol Sci. 2024 May 6;25(9):5050. doi: 10.3390/ijms25095050 (PMC11084877; doi:10.3390/ijms25095050)
Supplement: Supplementary file 1 [file ijms-25-05050-s001.zip › Table S8.pdf]

Table S8. Correlation analyses between the serum levels of adiponectin and clinical and laboratory parameters, separately for HVs and patients with MS.

	**Adiponectin (μg/mL)**			
	**HV (N=65)**		**MS (N=65)**	
**Variable**	**r**	**p**	**r**	**p**
Age (years)	0.09	0.4635	0.04	0.7370
BMI (kg/m^2)	-0.31	**0.0112**	-0.09	0.4803
WC (cm)	-0.50	**<0.0001**	-0.18	0.1474
MAP (mm Hg)	0.05	0.7130	0.09	0.4683
MetSSS	-0.60	**<0.0001**	-0.28	**0.0217**
Protein (g/L)	-0.25	**0.0457**	-0.13	0.2953
Albumin (g/L)	-0.34	**0.0061**	-0.13	0.2909
Glucose (mmol/L)	-0.05	0.6743	-0.10	0.4326
CRP (μg/mL)	-0.16	0.2046	-0.06	0.6458
IL-6 (pg/mL)	-0.13	0.3117	0.13	0.2997
Bilirubin (μmol/L)	-0.13	0.2908	-0.13	0.3084
AST (U/L)	-0.19	0.1298	-0.11	0.3973
ALT (U/L)	-0.33	**0.0075**	-0.17	0.1750
AP (U/L)	0.03	0.8321	0.16	0.2174
GGT (U/L)	-0.48	**0.0001**	-0.35	**0.0039**
LDH (U/L)	0.09	0.5002	0.17	0.1872
Urea (mmol/L)	-0.18	0.1412	0.00	0.9747
Urate (μmol/L)	-0.43	**0.0004**	-0.37	**0.0024**
Creatinine (μmol/L)	-0.37	**0.0026**	-0.30	**0.0170**
eGFR (mL/min/1.73 m^2)	0.01	0.9179	0.04	0.7754

Spearman correlation analyses were used to evaluate associations of the serum levels of adiponectin with clinical and laboratory parameters. *p*-values < 0.05 are considered statistically significant and are depicted in bold. ALT, alanine aminotransferase; AP, alkaline phosphatase; AST, aspartate aminotransferase; BMI, body mass index; CK, creatine kinase; CRP, C-reactive protein; eGFR, estimated glomerular filtration rate; GGT, gamma-glutamyl transpeptidase; HV, healthy volunteer; IL-6, interleukin 6; LDH, lactate dehydrogenase; MAP, mean arterial pressure; MetSSS, metabolic syndrome severity score; MS, metabolic syndrome patient; U, unit; WC, waist circumference.
