# Supplementary material for: Serum Levels of Adiponectin Are Strongly Associated with Lipoprotein Subclasses in Healthy Volunteers but Not in Patients with Metabolic Syndrome
Source: Int J Mol Sci. 2024 May 6;25(9):5050. doi: 10.3390/ijms25095050 (PMC11084877; doi:10.3390/ijms25095050)
Supplement: Supplementary file 1 [file ijms-25-05050-s001.zip › Table S9.pdf]

Table S9. Correlation analyses of serum levels of lipoprotein classes and subclasses with BMI, performed separately for the HVs and patients with MS.

	**BMI (kg/m²)**			
	**HV (N=65)**		**MS (N=65)**	
**Variable (mg/dL)**	**r**	**p-value**	**r**	**p-value**
**VLDL**				
VLDL-C	0.34	0.0060	-0.12	0.3346
VLDL1-C	0.38	0.0017	-0.12	0.3237
VLDL2-C	0.32	0.0093	-0.14	0.2552
VLDL3-C	0.29	0.0192	-0.10	0.4414
VLDL4-C	0.23	0.0630	-0.07	0.5627
VLDL5-C	0.19	0.1316	-0.06	0.6557
VLDL-FC	0.30	0.0138	-0.13	0.2884
VLDL1-FC	0.40	0.0009	-0.13	0.2926
VLDL2-FC	0.37	0.0022	-0.12	0.3273
VLDL3-FC	0.32	0.0086	-0.11	0.3705
VLDL4-FC	0.26	0.0366	-0.11	0.3732
VLDL5-FC	-0.02	0.9026	0.04	0.7800
VLDL-TG	0.32	0.0087	-0.11	0.3808
VLDL1-TG	0.34	0.0049	-0.10	0.4200
VLDL2-TG	0.31	0.0123	-0.15	0.2213
VLDL3-TG	0.28	0.0260	-0.14	0.2697
VLDL4-TG	0.23	0.0657	-0.07	0.5971
VLDL5-TG	0.07	0.5974	0.12	0.3222
VLDL-PL	0.32	0.0093	-0.12	0.3215
VLDL1-PL	0.41	0.0008	-0.11	0.4016
VLDL2-PL	0.35	0.0040	-0.13	0.2955
VLDL3-PL	0.29	0.0171	-0.13	0.3034
VLDL4-PL	0.28	0.0242	-0.08	0.5476
VLDL5-PL	0.23	0.0684	0.01	0.9530
VLDL-apoB	0.30	0.0166	-0.11	0.3647
**IDL**				
IDL-C	0.22	0.0850	-0.08	0.5321
IDL-FC	0.23	0.0620	-0.10	0.4171
IDL-TG	0.30	0.0154	-0.15	0.2185
IDL-PL	0.19	0.1326	-0.19	0.1304
IDL-apoB	0.13	0.3172	-0.04	0.7734
**LDL**				
LDL-C	0.05	0.6671	-0.10	0.4410
LDL1-C	-0.05	0.6809	-0.10	0.4303
LDL2-C	-0.19	0.1298	0.15	0.2335
LDL3-C	-0.02	0.8536	-0.09	0.4843
LDL4-C	0.25	0.0420	-0.22	0.0746
LDL5-C	0.18	0.1572	-0.14	0.2497
LDL6-C	-0.04	0.7402	0.05	0.6970
LDL-FC	-0.02	0.8493	-0.12	0.3267
LDL1-FC	-0.07	0.5995	-0.10	0.4134
LDL2-FC	-0.26	0.0352	0.19	0.1392
LDL3-FC	-0.17	0.1734	-0.05	0.7158
LDL4-FC	0.17	0.1704	-0.21	0.0922
LDL5-FC	0.15	0.2428	-0.18	0.1619
LDL6-FC	-0.10	0.4432	0.04	0.7672

LDL-TG	-0.01	0.9422	-0.05	0.7059
LDL1-TG	-0.06	0.6527	0.06	0.6165
LDL2-TG	-0.11	0.3805	0.15	0.2226
LDL3-TG	-0.18	0.1421	0.08	0.5488
LDL4-TG	0.17	0.1732	-0.13	0.3123
LDL5-TG	0.18	0.1502	-0.10	0.4136
LDL6-TG	-0.14	0.2630	0.09	0.4882
LDL-PL	0.04	0.7405	-0.09	0.4707
LDL1-PL	-0.07	0.5760	-0.06	0.6412
LDL2-PL	-0.18	0.1406	0.13	0.3008
LDL3-PL	-0.03	0.7888	-0.08	0.5403
LDL4-PL	0.26	0.0382	-0.23	0.0602
LDL5-PL	0.18	0.1536	-0.15	0.2327
LDL6-PL	-0.09	0.4927	0.04	0.7455
LDL-apoB	0.08	0.5281	-0.05	0.6750
LDL1-apoB	-0.08	0.5067	-0.03	0.7937
LDL2-apoB	-0.20	0.1189	0.17	0.1822
LDL3-apoB	-0.01	0.9310	-0.06	0.6075
LDL4-apoB	0.24	0.0544	-0.25	0.0409
LDL5-apoB	0.18	0.1418	-0.10	0.4251
LDL6-apoB	-0.02	0.8957	0.04	0.7217
**HDL**				
HDL-C	-0.42	0.0005	0.01	0.9360
HDL1-C	-0.38	0.0017	0.17	0.1746
HDL2-C	-0.36	0.0035	0.14	0.2778
HDL3-C	-0.31	0.0109	0.00	0.9806
HDL4-C	-0.04	0.7481	-0.09	0.4637
HDL-FC	-0.40	0.0009	-0.05	0.6748
HDL1-FC	-0.39	0.0013	0.01	0.9475
HDL2-FC	-0.37	0.0025	-0.01	0.9196
HDL3-FC	-0.32	0.0087	-0.14	0.2732
HDL4-FC	-0.05	0.6733	-0.13	0.2964
HDL-TG	-0.10	0.4510	-0.10	0.4144
HDL1-TG	-0.30	0.0145	0.00	0.9909
HDL2-TG	-0.10	0.4475	-0.02	0.9014
HDL3-TG	0.04	0.7645	-0.05	0.7036
HDL4-TG	0.18	0.1561	-0.21	0.0944
HDL-PL	-0.45	**0.0002**	0.00	0.9697
HDL1-PL	-0.38	0.0018	0.15	0.2184
HDL2-PL	-0.37	0.0024	0.13	0.3171
HDL3-PL	-0.32	0.0086	0.00	0.9854
HDL4-PL	-0.09	0.4660	-0.11	0.3627
HDL-apoA-I	-0.45	**0.0001**	-0.13	0.3065
HDL1-apoA-I	-0.39	0.0014	0.11	0.3766
HDL2-apoA-I	-0.48	**<0.0001**	0.11	0.3859
HDL3-apoA-I	-0.32	0.0085	0.02	0.8643
HDL4-apoA-I	-0.02	0.8894	-0.17	0.1790
HDL-apoA-II	-0.20	0.1169	-0.15	0.2435
HDL1-apoA-II	-0.41	0.0008	0.02	0.8456
HDL2-apoA-II	-0.36	0.0030	0.05	0.6862
HDL3-apoA-II	-0.12	0.3599	-0.05	0.6749
HDL4-apoA-II	0.04	0.7599	-0.20	0.1190

Spearman correlation analyses were used to evaluate the associations between the serum levels of lipoproteins and BMI. p-values < 0.0005 are considered statistically significant after a Bonferroni correction for multiple testing and are depicted in bold. ApoA-I, apolipoprotein A-I; apoA-II, apolipoprotein A-II; apoB, apolipoprotein B; BMI,

body mass index; C, cholesterol; FC, free cholesterol; HV, healthy volunteer; HDL, high-density lipoprotein; IDL, intermediate-density lipoprotein; IL-6, interleukin-6; LDL, low-density lipoprotein; MS, metabolic syndrome patient; PL, phospholipid; r, Spearman's correlation coefficient; TG, triglyceride; VLDL, very low-density lipoprotein.
